# *NTRK3* Is a Potential Tumor Suppressor Gene Commonly Inactivated by Epigenetic Mechanisms in Colorectal Cancer

**DOI:** 10.1371/journal.pgen.1003552

**Published:** 2013-07-11

**Authors:** Yanxin Luo, Andrew M. Kaz, Samornmas Kanngurn, Piri Welsch, Shelli M. Morris, Jianping Wang, James D. Lutterbaugh, Sanford D. Markowitz, William M. Grady

**Affiliations:** 1 Department of Colorectal Surgery, The Sixth Affiliated Hospital, Sun Yat-Sen University, Guangzhou, P.R. China; 2 Clinical Research Division, Fred Hutchinson Cancer Research Center, Seattle, Washington, United States of America; 3 Research and Development Service, VA Puget Sound Health Care System, Seattle, Washington, United States of America; 4 Department of Medicine, University of Washington School of Medicine, Seattle, Washington, United States of America; 5 Tumor Biology Research Unit and Department of Pathology, Faculty of Medicine, Prince of Songkla University, Hat Yai, Songkhla, Thailand; 6 Division of Medical Genetics, University of Washington Medical School, Seattle, Washington, United States of America; 7 Department of Medicine and Ireland Cancer Center, Case Western Reserve University School of Medicine and Case Medical Center, Cleveland, Ohio, United States of America; Centre for Cancer Biology, SA Pathology, Australia

## Abstract

*NTRK3* is a member of the neurotrophin receptor family and regulates cell survival. It appears to be a dependence receptor, and thus has the potential to act as an oncogene or as a tumor suppressor gene. NTRK3 is a receptor for NT-3 and when bound to NT-3 it induces cell survival, but when NT-3 free, it induces apoptosis. We identified aberrantly methylated *NTRK3* in colorectal cancers through a genome-wide screen for hypermethylated genes. This discovery led us to assess whether *NTRK3* could be a tumor suppressor gene in the colon. *NTRK3* is methylated in 60% of colon adenomas and 67% of colon adenocarcinomas. *NTRK3* methylation suppresses *NTRK3* expression. Reconstitution of NTRK3 induces apoptosis in colorectal cancers, if NT-3 is absent. Furthermore, the loss of *NTRK3* expression associates with neoplastic transformation *in vitro* and *in vivo*. We also found that a naturally occurring mutant *NTRK3* found in human colorectal cancer inhibits the tumor suppressor activity of *NTRK3*. In summary, our findings suggest *NTRK3* is a conditional tumor suppressor gene that is commonly inactivated in colorectal cancer by both epigenetic and genetic mechanisms whose function in the pathogenesis of colorectal cancer depends on the expression status of its ligand, NT-3.

## Author Summary

NTRK3 is a neurotrophin receptor and appears to be a dependence receptor in certain tissues. *NTRK3* has been previously shown to be an oncogene in breast cancer and possibly hepatocellular carcinoma. Through a genome-wide methylation screen, we unexpectedly found that *NTRK3* is commonly methylated in colorectal cancers but not in normal colon samples, which led us to assess whether *NTRK3* could be a tumor suppressor gene in the colon. We now demonstrate that *NTRK3* is frequently methylated in colorectal adenomas and cancers. Induced NTRK3 expression in the absence of its ligand, NT-3, causes apoptosis and suppresses *in vitro* anchorage-independent colony formation and *in vivo* tumor growth. Reintroduction of NT-3 releases colon cancer cells from NTRK3-mediated apoptosis, which is consistent with NTRK3 being a dependence receptor in the colon. Finally, somatic mutations of *NTRK3* have been observed in primary human colorectal cancer. We provide evidence that a subset of these mutations inactivate tumor suppressor activities of *NTRK3*. These findings suggest that *NTRK3* is a conditional tumor suppressor gene in the colon that is inactivated by both genetic and epigenetic mechanisms and whose function in the pathogenesis of colorectal cancer depends on the expression status of its ligand, NT-3.

## Introduction

Colorectal cancer (CRC) arises through the accumulation of gene mutations and epigenetic alterations that result in the transformation of normal colon epithelial cells into adenocarcinomas [1]. One of the most common epigenetic changes observed in CRC is the aberrant methylation of CpG islands in the promoter region of genes. Aberrant CpG island methylation is associated with gene silencing and can inactivate tumor suppressor genes in the colon, which promotes tumor formation through the deregulation of various cellular processes including proliferation and apoptosis, among others [1], [2].

In order to identify methylated tumor suppressor genes that play a role in the formation of CRC, we conducted a genome-wide screen for methylated genes in colorectal cancers and matched normal colon epithelium tissue samples. Through this screen, we found a set of novel methylated genes, which included neurotrophin tyrosine kinase receptor 3 (*NTRK3*), a gene that has been found to be hypermethylated in esophageal adenocarcinoma [3]. The identification of methylated *NTRK3* in CRC was unexpected given that *NTRK3* has been shown to be an oncogene in breast cancer and possibly hepatocellular carcinoma [4], [5]. However, *NTRK3* has also been shown to be a tumor suppressor gene in neuroblastomas [6]. Thus, our findings raised the question of whether *NTRK3* acts as an oncogene or tumor suppressor gene in the pathogenesis of CRC.

*NTRK3* is a member of the NTRK neurotrophin receptor family, which includes *NTRK1 (TRKA)*, *NTRK2 (TRKB)* and *NTRK3 (TRKC)*. NTRK family members and their ligands, nerve growth factor (NGF), brain-derived neurotrophic factor (BDNF), neurotrophin-3 (NT-3) and NT4/5, are crucial to the development of the nervous system and have poorly defined roles in other tissues [7]. NTRK1 is the receptor for NGF, and NTRK2 preferentially binds BDNF and NT4/5. NT-3 is the only known physiologically relevant ligand for NTRK3. The NTRKs have been shown to play oncogenic roles in certain cancers, such as breast cancer and liver cancer [8]. For example, a fusion of *ETV6* (ETS translocation variant 6) to *TRKC* leads to the constitutive activation of TRKC tyrosine kinase, which promotes tumor formation and progression in human breast carcinoma [9].

However, rather than being classic tyrosine kinase receptors, recent data suggests that NTRK1 and NTRK3 may be dependence receptors [6], [10], [11]. Dependence receptors are characterized by their ability to induce opposing biological effects depending on the availability of their ligands. In the presence of the receptor's ligand, a positive cellular differentiation or survival signal is transduced, whereas lack of the ligand results in cleavage of a death-domain peptide and induction of apoptosis [12]. Indirect support for the role of *NTRK3* as a dependence receptor and conditional tumor suppressor gene is provided by the observation that *NTRK3* is a favorable-prognostic factor in a variety of cancers, such as melanoma [13] and medulloblastomas [14]. These findings suggested that *NTRK3* might likewise serve as a conditional tumor suppressor gene in colorectal cancer.

With regards to the possibility that *NTRK3* could act as a tumor suppressor gene in the colon rather than as an oncogene, somatic inactivating mutations of *NTRK3* have been identified in CRC [15], as well as in other cancers including breast, lung, and pancreatic [16]. These mutations are missense mutations that are predicted to inhibit the function of NTRK3 [16] (See **Table S1**). Thus, the discovery of mutant, as well as methylated, *NTRK3* in CRC suggested the possibility of *NTRK3* being a CRC tumor suppressor gene. Consequently, we carried out a series of studies to determine the effect of aberrant DNA methylation on the expression of *NTRK3* and to determine if NTRK3 had oncogene or tumor suppressor activities in colorectal cancer cell lines.

## Results

### Aberrant methylation of *NTRK3* is common in colorectal adenomas and cancers

DNA from CRCs and normal colon mucosa samples was subjected to analysis using Infinium HumanMethylation450 BeadChip arrays (Illumina). After filtering the data as described previously [3], we identified a number of genes that were aberrantly methylated in the CRCs. One of these methylated genes was *NTRK3*, which was methylated in all the CRCs and in none of the normal colon epithelium samples. In light of the preferential methylation of *NTRK3* in CRCs and because of its role as a neurotrophin receptor, which suggested it could have a functional role in the formation of colorectal cancer, we carried out a series of studies to further assess the effect of *NTRK3* methylation on CRCs.

**Figure 1:**
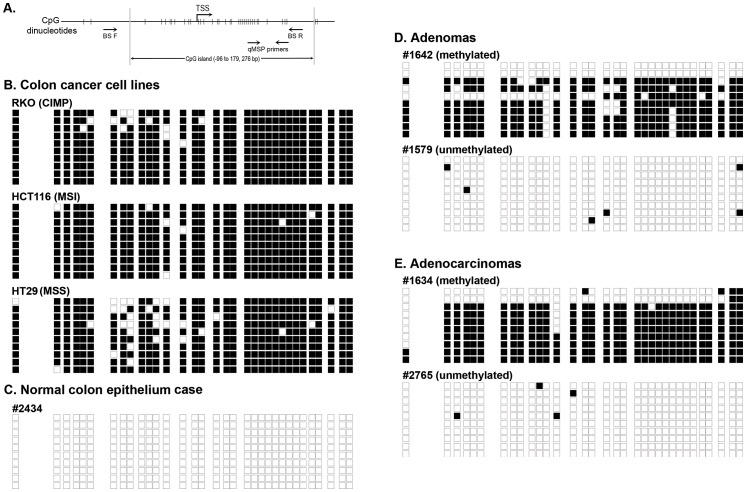
Bisulfite sequencing results of representative methylated and unmethylated samples as determined by the *NTRK3* MethyLight (qMSP) assay. The black boxes indicate methylated CpG dinucleotides, and the white boxes represent unmethylated CpG dinucleotides. Each row depicts the sequencing results for a single clone. **A.** Schematic diagram of the 5′ end of the *NTRK3* gene showing the location of the qMSP and bisulfite sequencing primers (TSS: transcriptional start site). **B.** Results of bisulfite sequencing of DNA from colon cancer cell lines representative of different molecular subtypes: RKO is a CpG island methylator phenotype (CIMP) cell line, HCT116 is microsatellite unstable (MSI) line, and HT29 is a microsatellite stable (MSS) line. These cell lines have dense methylation of *NTRK3*. **C.** Representative case of normal colon, which carries unmethylated *NTRK3*. **D. and E.** Representative cases of adenomas and adenocarcinomas that carry either methylated or unmethylated *NTRK3* are shown.

The promoter region of *NTRK3* (NM_002530) contains a dense CpG island located from nucleotides −96 to +179 relative to the transcription start site (TSS; **Figure 1A**). After observing methylated *NTRK3* in the colorectal cancers run on the HumanMethylation450 arrays, we assessed the methylation status of *NTRK3* in a second independent set of normal colon mucosa, colon adenomas, and CRCs using a quantitative methylation-specific PCR assay (qMSP; MethyLight) designed to assess the promoter region of *NTRK3*. We first established that a Percent of Methylated Reference (PMR) threshold of 13.7% had a specificity of ∼90% for cancer vs. normal tissue. Using this PMR threshold, we detected *NTRK3* promoter methylation in 67% of colorectal cancers (N = 76) (See **Text S1** for methods used to determine optimal PMR. **Figure S11 and Table S4**). Using this same PMR threshold, *NTRK3* promoter methylation was found in 60% of adenomas (N = 55) and 10% of the normal colon samples (N = 98; normal versus cancer: *p*<0.0001; normal versus adenoma: *p*<0.0001). The frequency of normal colon mucosa cases adjacent to CRC with *NTRK3* promoter methylation did not differ significantly from that observed in the normal mucosa of cancer-free individuals (**Table 1**). We also assessed the status of *NTRK3* in a panel of colon cancer cell lines (N = 9) and found that all the cell lines had methylated *NTRK3* using the 13.7% PMR threshold.

**Table 1:** *NTRK3* promoter methylation in colorectal adenocarcinoma, adenoma and normal colon epithelium.

Description of Sample	N	Methylated cases (percentage, %)
		PMR = 13.7¶
**Adenocarcinoma**	**76**	**50 (65.8%)**
Stage I or II	18	12 (66.7%)
Stage III	25	17 (68.0%)
Stage IV	14	12 (85.7%)
Unknown stage	19	9 (47.4%)
**Adenoma**	**55**	**33 (60.0%)**
Early adenoma	25	13 (52.0%)
Advanced adenoma	27	19 (70.4%)
Unknown stage	3	1 (33.3%)
**Normal colon epithelium** ‡	**98**	**9 (9.18%)**
CRC patients	46	4 (8.70%)
Cancer-free patients	52	5 (9.62%)

¶ Methylation was scored as being present in a case if the PMR >13.7. The PMR threshold was optimized by using ROC analysis on cancer and normal samples with an area under the curve (AUC) of 0.843.

‡ “Cancer-free patients” indicates that the patient did not have colorectal cancer.

In addition, we performed bisulfite sequencing of the promoter region of *NTRK3* in representative cases of normal colon epithelium, adenomas, and adenocarcinomas (5 samples/group) and correlated these results with those of the *NTRK3* qMSP assay. The bisulfite sequencing results correlated well with the qMSP results (**Figure 1B–E**).

### Methylation of *NTRK3* is independent of CIMP status, MSI status, *KRAS* mutations and *BRAF^V600E^* mutations in colorectal cancer

Colorectal cancer can be classified into molecular classes, which include the Microsatellite Unstable (MSI), Chromosome Unstable (CIN, also known as Microsatellite Stable, MSS), and CpG Island Methylator Phenotype (CIMP) [1]. These classes of CRC appear to have unique pathogenic mechanisms that give rise to the CRCs. We assessed the association of methylated *NTRK3* with these classes of CRC and with mutations that are commonly found in CRC. As shown in **Table 2**, methylated *NTRK3* is more frequent in tumors in women, and is independent of CIMP and MSI status, as well as *KRAS*, *BRAF^V600E^*, *TP53*, *PIK3CA* or *APC* mutations. In addition, as shown in **Figure S1**, methylated *NTRK3* appears to be independent of other genes that are frequently methylated in CRC, such as *MLH1*, *CDKN2A/p16* or *RASSF1A*.

**Table 2:** Clinical, pathological, and molecular characteristics of CRCs that carry methylated *NTRK3* and unmethylated *NTRK3*.

		NTRK3		
		Methylated	Unmethylated	*p*
**All**		**25**	**7**	
**Age**				**1**
>60		12	4	
≤60		13	3	
**Gender**				**0.03181**
Male		6	5	
Female		19	2	
**Stage**				**1**
I or II		8	2	
III or IV		17	5	
**Tumor Location**				**1**
Colon		22	6	
Rectum		3	1	
**CIMP**				**0.1497**
Yes		8	0	
No		17	7	
**MSI**				**0.5896**
Yes		4	2	
No		21	5	
**KRAS**				**0.6833**
Wild-type	15	5		
Mutant		10	2	
**BRAF**				**0.2964**
Wild-type	19	7		
Mutant		6	0	
**APC**				**0.2964**
Wild-type	22	5		
Mutant		3	2	
**TP53**				**0.6479**
Wild-type	18	4		
Mutant		7	3	
**PIK3CA**				**0.5523**
Wild-type	21	7		
Mutant		4	0	

### Methylation of *NTRK3* silences *NTRK3* expression

**Figure 2:**
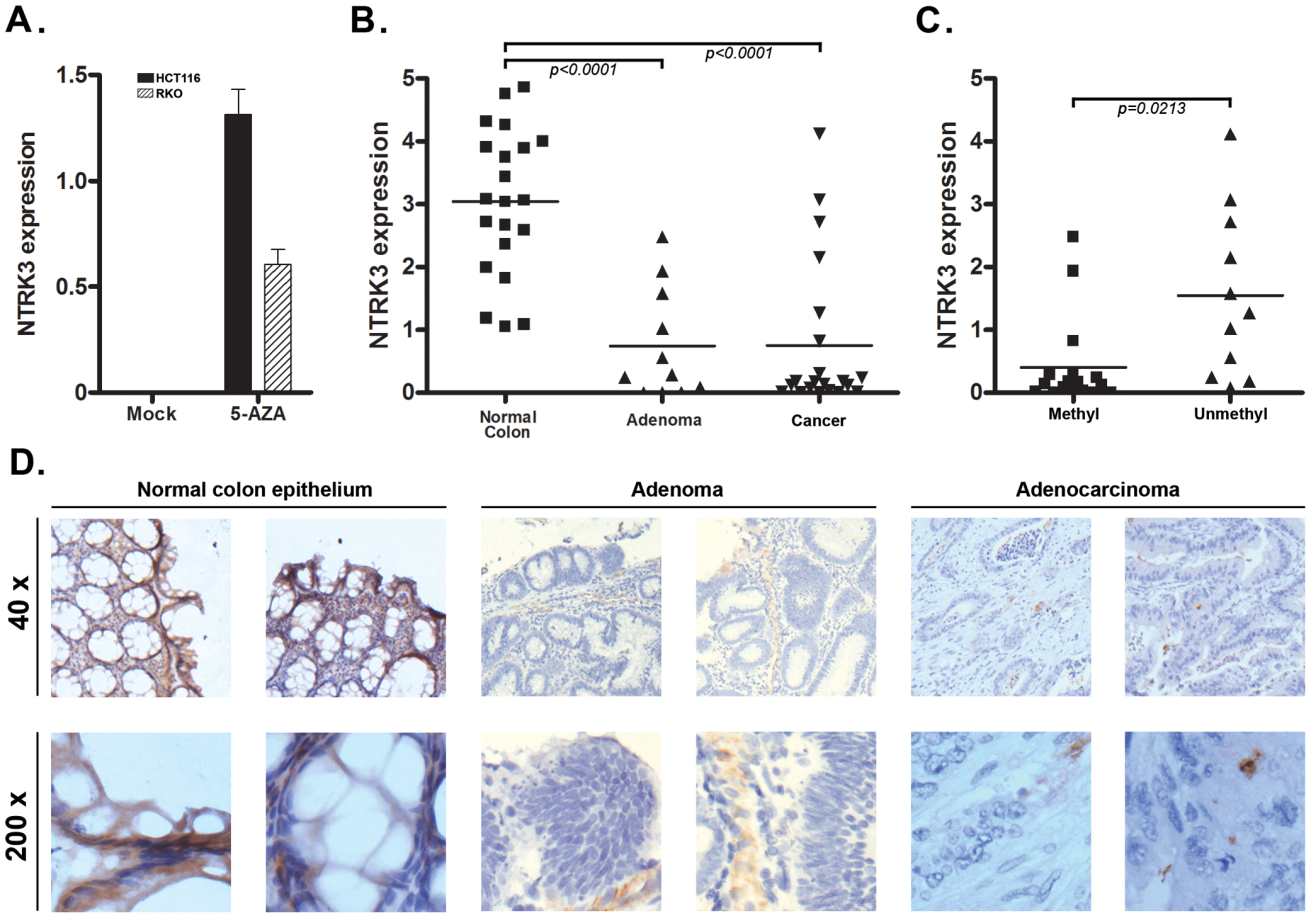
*NTRK3* methylation correlates with reduced *NTRK3* mRNA expression. **A.** *NTRK3* mRNA expression after treatment with 5-aza-2′-deoxycytidine (5-AZA). Colon cancer cell lines HCT116 and RKO, which carry methylated *NTRK3*, have no detectable *NTRK3* mRNA expression when treated with the vehicle only (Mock). The expression of *NTRK3* is increased significantly after 5-AZA treatment in both lines. **B.** Expression of *NTRK3* mRNA in primary normal colon mucosa, colon adenomas, and colon adenocarcinomas (cancer). When compared to normal colon mucosa (average expression = 3.04±0.25, N = 21), *NTRK3* expression is significantly lower in the colon adenomas (average expression = 0.75±0.27, N = 11) and cancers (average expression = 0.75±0.27, N = 21), presumably related to the high prevalence of colorectal adenomas and adenocarcinomas that carry methylated *NTRK3* (*P*<0.0001, one way ANOVA). **C.** *NTRK3* mRNA expression is higher in the adenomas and cancers that carry unmethylated *NTRK3* (0.40±0.17, N = 17) compared to tumors that carry methylated *NTRK3* (1.55±0.40, N = 11; *P* = 0.0213, 2-sided student *t* test). (The units on the Y-axis in A–C are relative expression units.) **D.** NTRK3 immunostaining of representative cases of normal colon mucosa, adenomas and adenocarcinomas (40×, upper panels; 200×, lower panels). NTRK3 is expressed in the normal colon epithelium cells, however, NTRK3 is reduced or absent in the adenomas or adenocarcinomas. The adenocarcinomas and adenomas shown all carry methylated *NTRK3*.

As mentioned above, we noticed that all nine of the colon cancer cell lines analyzed carried methylated *NTRK3*. Consistent with methylation silencing *NTRK3* expression, we did not detect *NTRK3* mRNA expression in any of these cell lines. Next, the CRC cell lines RKO and HCT116, which carry methylated *NTRK3*, were treated with 5-aza-2′-deoxycytidine (5-AZA), which inhibits DNA methyltransferase1 (DNMT1), to determine if demethylation of the *NTRK3* promoter would induce *NTRK3* expression. Following 5-AZA treatment, *NTRK3* mRNA expression was induced in both HCT116 and RKO cells (**Figure 2A**). We next assessed *NTRK3* mRNA expression in normal colon mucosa samples, colorectal adenomas, and primary colon adenocarcinomas. *NTRK3* mRNA expression was significantly lower in colorectal adenocarcinomas and adenomas as compared to the matched normal colon mucosa, which carried unmethylated *NTRK3* (**Figure 2B**). Moreover, the expression of *NTRK3* was significantly higher in the primary colon tumors that carry unmethylated *NTRK3* compared to the tumors that carry methylated *NTRK3* (**Figure 2C**).

We also assessed NTRK3 protein expression in normal colon mucosa and in adenomas and colorectal cancer by immunostaining. The normal colon mucosa showed heterogeneous membrane and cytoplasmic staining using an anti-NTRK3 monoclonal antibody, whereas almost no expression was detected in most adenoma and cancer cases (N = 30). Among the 20 adenoma and cancer samples, only 6 samples showed weak or moderate NTRK3 expression, while 9 out of 10 normal samples showed strong or moderate NTRK3 expression (**Figure 2D**). Taken together, these data provide support for the aberrant methylation of the *NTRK3* promoter silencing NTRK3 expression in colon neoplasms.

### Expression of NT-3 in colon cancer cell lines and primary tissues

**Figure 3:**
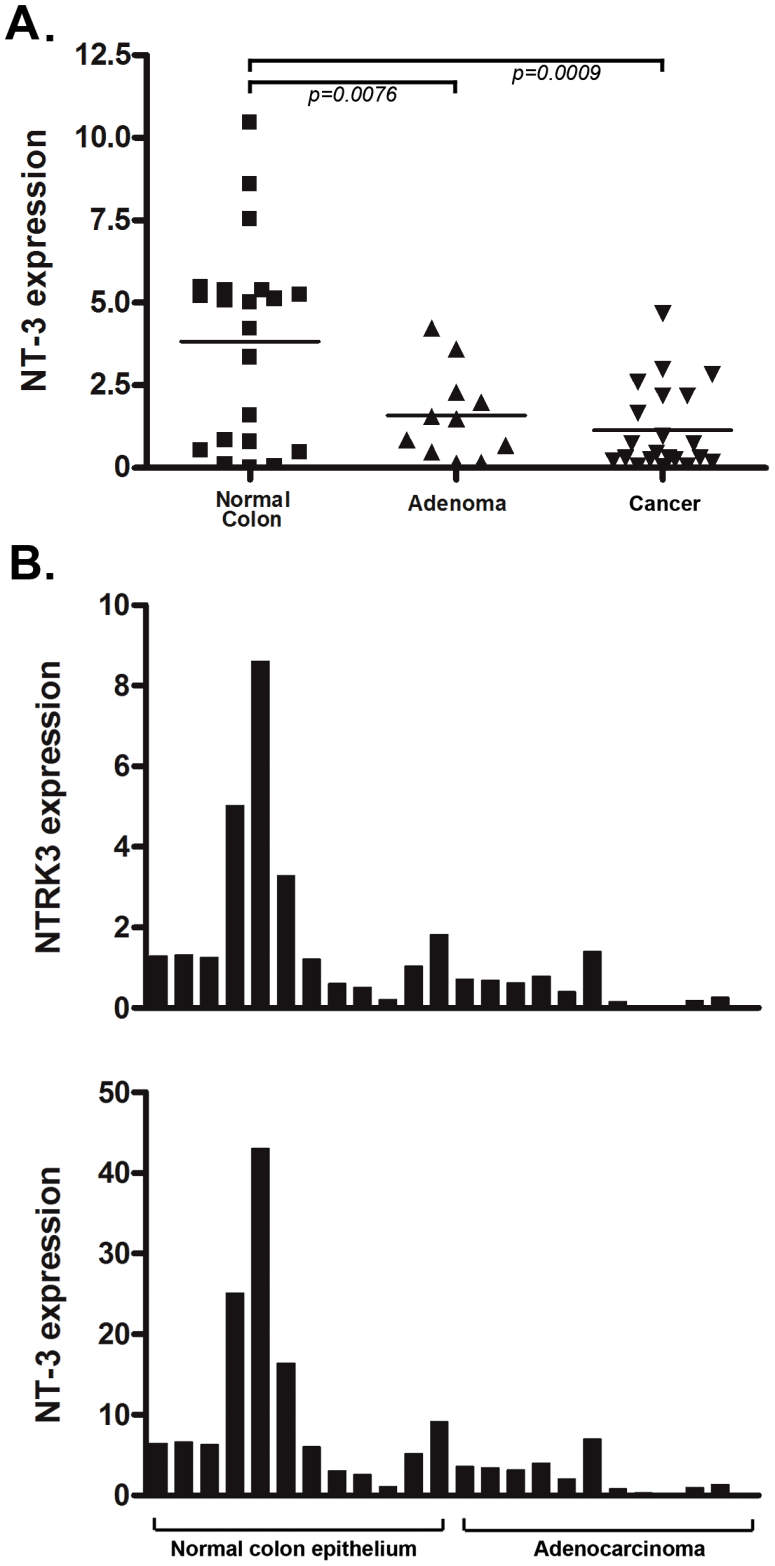
*NT3* expression and the relationship of *NTRK3* and *NT3* mRNA expression in primary colon tissues. **A.** *NT3* mRNA expression is significantly lower in both the adenoma and adenocarcinoma samples compared to the matched normal colon epithelium (both *p*<0.01) (Please see methods for units on Y axis). **B.** There is a direct correlation between *NT3* and *NTRK3* expression in normal colon and adenocarcinomas (r^2^ = 0.81, Pearson's correlation *P*<0.0001). The units on the Y-axis are relative expression units and each bar on the X-axis represents one sample. The bar graphs are aligned so that the samples correspond in the upper and lower graphs.

Because NTRK3 has been shown to function as a dependence receptor in certain tissues, we also assessed the expression of NTRK3's ligand, NT-3, in CRC cell lines and primary CRCs. NTRK3's preferred ligand is NT-3, and NT-3 has been shown to inhibit NTRK3 mediated apoptosis and to induce NTRK3-mediated activation of signaling pathways involved in cell proliferation, apoptosis and motility [6], [17]. Therefore, we assessed the expression levels of NT-3 in the panel of colon cancer cell lines previously assessed for methylated *NTRK3*. No NT-3 expression was detected in RKO, HCT116, FET, Vaco400 and HT-29, whereas NT-3 was expressed at a low (although relatively high level in relation to the other CRC cell lines) in SW480. NT-3 expression was present at low levels in Lovo, LS174T, and AAC1/SB10 (**Figure S2A**). We next assessed the expression of NT-3 in primary CRC tissues and in matched normal colon mucosa specimens. NT-3 expression was significantly lower in the CRC's when compared to the normal colon (**Figure 3A**). Interestingly, we found a direct correlation between NT-3 expression and NTRK3 expression in the normal colon and in the CRC's (r^2^ = 0.81, Pearson's correlation *P*<0.0001), suggesting that the presence of NT-3 relieved the selective pressure to silence NTRK3 (**Figure 3B**).

In order to determine the mechanism responsible for loss of NT-3 expression, we assessed the methylation status of the promoter region of *NT3* using an *NT3* MSP assay and correlated these results with the *NT3* mRNA expression levels. We found that the colon cancer cell lines lacking *NT3* expression have aberrantly methylated *NT3*, whereas those that express *NT3* mRNA carry unmethylated *NT3* (**Figure S2C**). We also found that 5-AZA treatment of two cell lines that carry methylated *NT3*, HCT116 and RKO, induces the expression of *NT3* (**Figure S2B**). Therefore, we conclude that the methylation of *NT3* can repress the expression of *NT3*.

### NTRK3 is a dependence receptor in CRC

**Figure 4:**
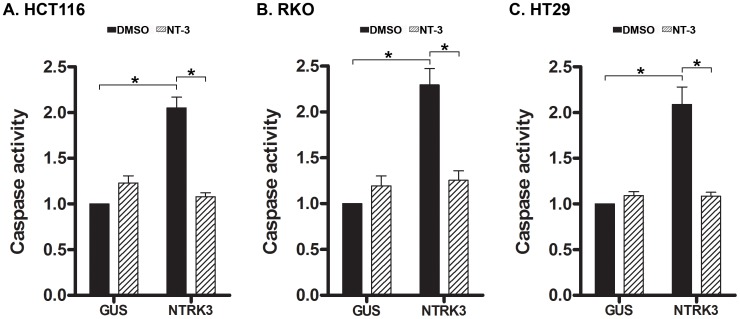
Assessment of normalized caspase 3 and 7 activity after reconstitution of *NTRK3* in HCT116 (A), RKO (B) and HT29 (C) cells. NTRK3 induces caspase activity in HCT116 (MSI), RKO (CIMP) and HT29 (MSS) cells, and NT-3 (100 ng/mL) inhibits this effect in all three cell lines. DMSO is the vehicle control for NT-3, and GUS is the control vector (pDEST27-GUS) used to normalize for nonspecific effects of the transfection on caspase activity. The caspase activity in the GUS transfected cells treated with DMSO was used to normalize the results from the other experimental groups. The asterisks indicate statistically significant differences, *p*<0.05 as determined by a 2-sided Mann-Whitney rank sum test.

NTRK3 has been shown to be a dependence receptor in certain tumors and can trigger caspase-based apoptosis when not bound by NT-3 [6], [10]. In the presence of NT-3, NTRK3 induces differentiation, guidance or survival in neurons; however, NTRK3 can alternatively induce apoptotic cell death in the absence of NT-3 in neuroblastoma cells and presumably other cell types [6]. The dependence receptor aspect of the biological effects of NTRK3 suggests it has the potential to be either an oncogene or a tumor suppressor gene, depending on the presence of NT-3. In order to assess the effect of NTRK3 on colorectal cancer, *NTRK3* was transfected into the HCT116 (MSI), RKO (CIMP) and HT29 (CIMP/MSS) cell lines, which lack *NTRK3* and *NT3* mRNA expression (**Figure S3A and B**). In these cell lines, *NTRK3* reconstitution increased caspase activity by 2–3 fold compared to the control vector transfected cells. Furthermore, the addition of NT-3 (100 ng/mL) suppressed apoptosis induced by *NTRK3* reconstitution (**Figure 4A, B and C** **)**. These results were confirmed using an independent assay that assesses apoptosis by detecting apoptosis specific DNA:histone complexes (Cell Death Detection Assay (Roche); **Figure S4 A, B and C**).

### Somatic *NTRK3* mutations occur in primary colorectal cancer and can inactivate NTRK3

**Figure 5:**
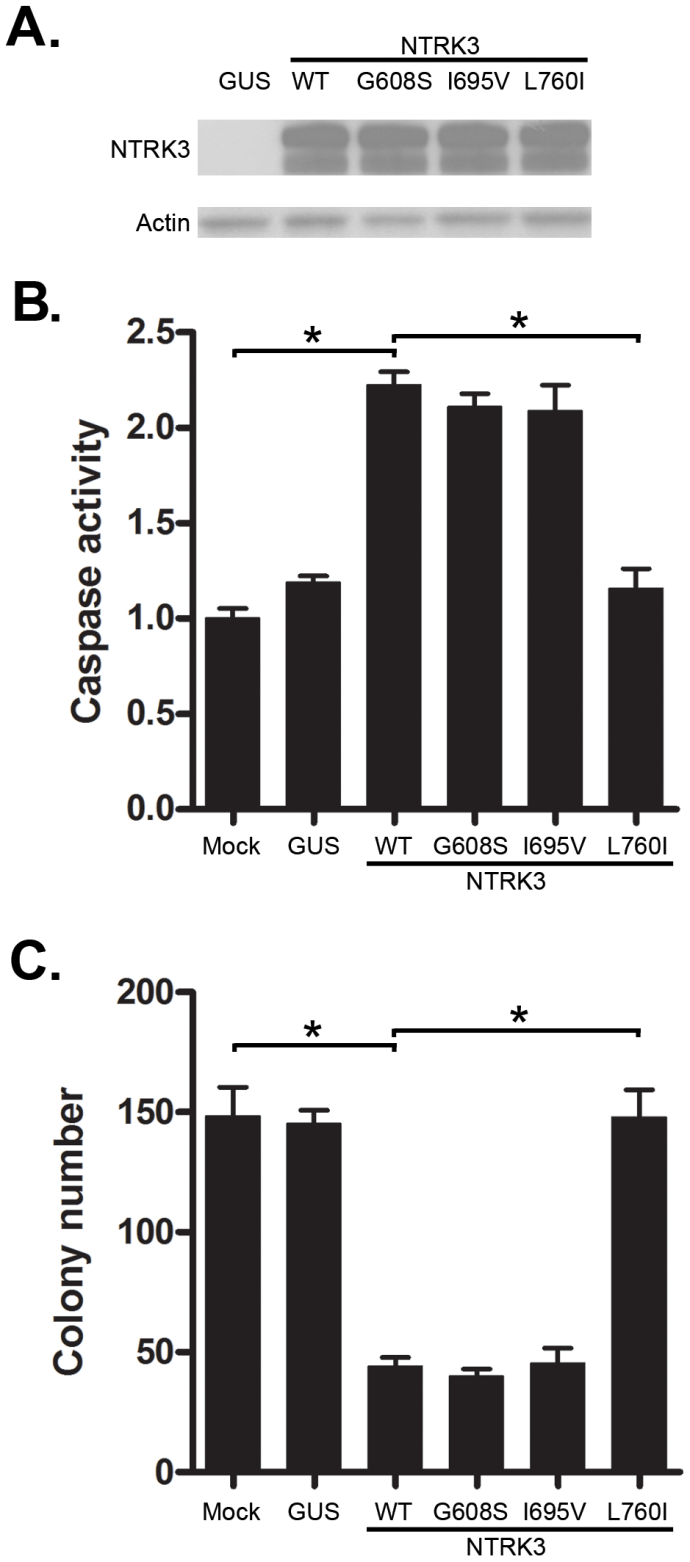
Assessment of caspase activity and colony formation after reconstitution with mutant *NTRK3* in RKO. The three mutant *NTRK3* constructs contain *NTRK3* mutants found in primary human CRC. **A.** The expression of reconstituted wild-type *NTRK3* (*WT*), *NTRK3-G608S (G608S)*, *NTRK3-I695V (I695V)* and *NTRK3-L760I (L760I)* was confirmed by western blotting. **B.** Apoptosis was assessed by normalized caspase 3 and 7 activity in the RKO cell line 48 hours after transfection with *WT*, *G608S*, *I695V* and *L760I NTRK3* constructs. **C.** Soft agar colony formation was assessed in stably-transfected RKO cells after 2 weeks. Results are plotted as the mean colony numbers from three independent experiments. *L760I* did not induce apoptosis (**B**) or suppress colony formation (**C**), whereas *WT*, *G608S* and *I695V* did. Thus, the *G608S* and *I695V* mutations appear to be passenger mutations. GUS was used as the control vector to normalize for nonspecific effects of the transfection on apoptosis. The asterisks indicate statistically significant differences, p<0.05 as determined by a 2-sided Mann-Whitney rank sum test.

Somatic mutations of *NTRK3* have been identified in primary colorectal cancers [15]. In order to determine the effect of the mutant *NTRK3* genes on the behavior of colorectal cancers, we constructed plasmids that express the following *NTRK3* mutants: *NTRK3-G608S*, *NTRK3-I695V* and *NTRK3-L760I* [15]. The mutant *NTRK3* constructs were then transfected into the CRC cell line RKO. Transfection of *NTRK3-L760I* into the RKO cells did not induce apoptosis (**Figure 5B**), but the wild-type *NTRK3*, *NTRK3-G608S* or *NTRK3-I695V* alleles did induce apoptosis. Moreover, inhibition of colony formation by NTRK3 was not induced by *NTRK3-L760I*, but was induced by *NTRK3-G608S* and *NTRK3-I695V* (**Figure 5C**). These findings demonstrate that *NTRK3-L760I* inactivates *NTRK3* with regards to its apoptosis and colony formation ability. However, the other mutant alleles do not affect the function of NTRK3 and are presumably passenger mutations. (Of note, the mutation status of all the constructs was confirmed by direct sequencing, see **Figure S5**). Also, we did not observe any change in NT-3 expression after transfection with the wild-type or mutant *NTRK3* constructs. These findings suggest *NTRK3* is a tumor suppressor gene in the colon that can be inactivated by both epigenetic and genetic mechanisms. The identification of both methylated *NTRK3* and inactivating *NTRK3* mutations in colorectal cancers provides evidence that inactivation of *NTRK3* promotes tumor formation in the colon.

### Loss of NTRK3 leads to perturbed MAPK signaling pathway

**Figure 6:**
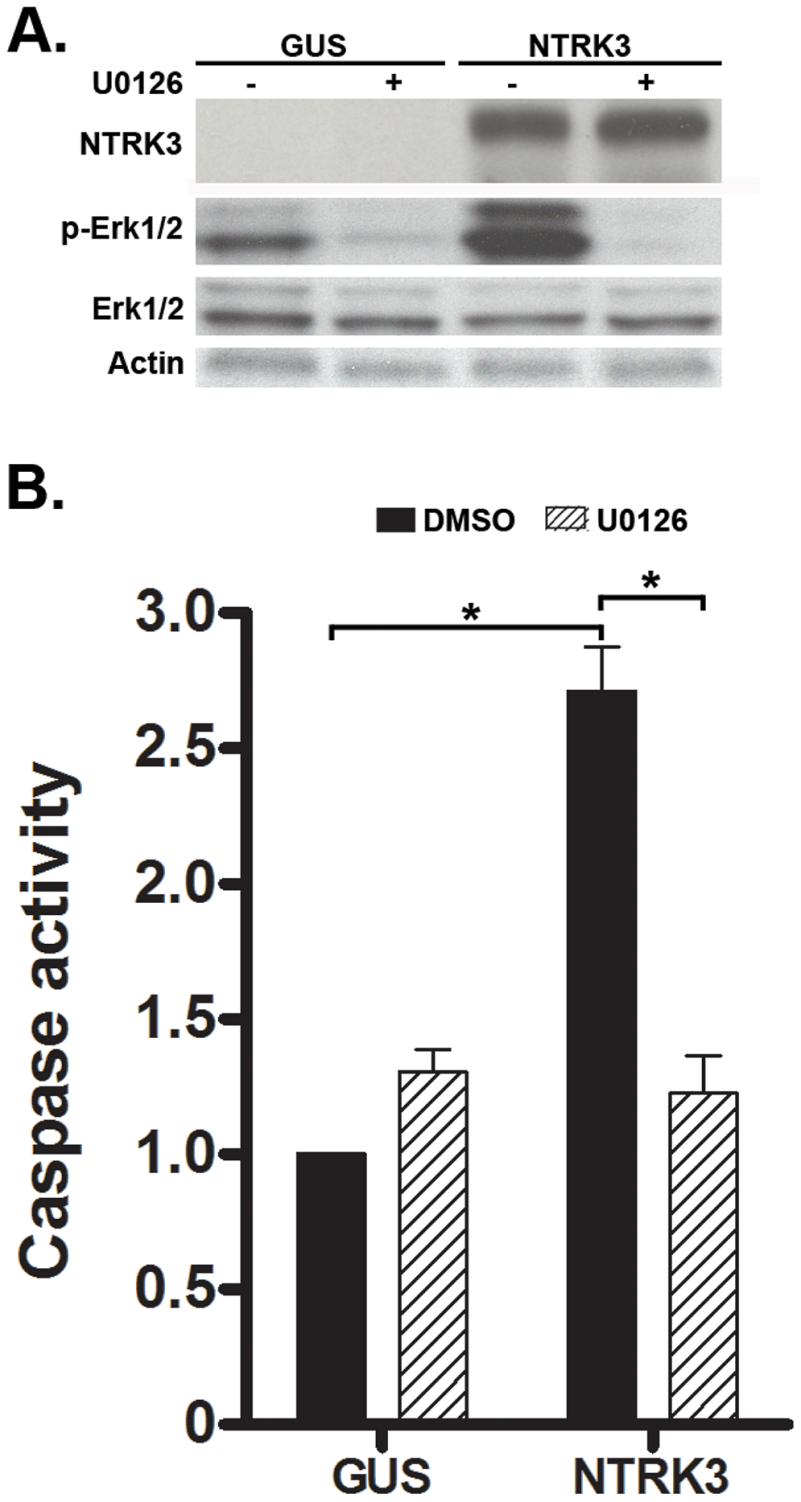
NTRK3 expression induces phosphorylation of Erk1/2 (Thr202/Tyr204), and the inhibition of phosphorylation of Erk1/2 correlates with decreased apoptosis induced by NTRK3. HCT116 cells were transfected with either *GUS* or *NTRK3*, and then 24 hours after the transfection, were treated with the MAPK inhibitor U0126 (10 µM) for an additional 24 hours. **A.** NTRK3 mediated phosphorylation of Erk1/2 is inhibited by U0126. **B.** U0126 reverses the caspase activation induced by NTRK3 expression in HCT116 cells. DMSO was used as a vehicle control. The asterisks indicate statistically significant differences (*p*<0.05; 2-sided Mann-Whitney rank sum test). Caspase activity was normalized to the *GUS* vector transfected HCT116 cells treated with DMSO.

As shown in the experiments above, *NTRK3* can act as a tumor suppressor gene in colon cancer cell lines and can induce apoptosis in CRC cell lines through the activation of caspase 3 or caspase 7. We next assessed the signaling pathways that are affected by NTRK3, which have been shown to include the MAPK/Erk, NF-κB and PI3K/Akt pathways, to determine if they may be mediating NTRK3 induced apoptosis in the colon cancer cell lines [18]. We initially assessed the activation status of the MAPK/Erk pathway in the HCT116 and RKO cell lines. HCT116 and RKO cells reconstituted with NTRK3 show increased activation of the MAPK/Erk pathway as determined by increased phospho-Erk1/2 (p-Erk1/2) expression. This increase in p-Erk1/2 was accompanied by increased caspase3/7 activity. The NTRK3-induced apoptosis was inhibited by the MAPK inhibitor U0126 (**Figure 6A and B**). In order to confirm that increased p-Erk1/2-induced apoptosis was specific to NTRK3, we used 16% FBS as an extracellular stimulus to induce increased p-Erk1/2. Not surprisingly, cells treated with 16% FBS showed significant increased p-Erk1/2, which was not accompanied by increased apoptosis (**Figure S6**). Since the addition of NT-3 inhibited apoptosis induced by NTRK3 expression, we assessed whether the introduction of NT-3 affected the activation status of the MAPK/Erk pathway. Interestingly, the addition of NT-3 decreased NTRK3 protein expression and decreased p-Erk1/2 levels (**Figure S7**). These findings suggest that at least part of *NTRK3*'s pro-apoptotic effects occur through the MAPK signaling pathway in colon cancer cell lines.

In light of the prior reports implicating a fusion gene involving *NTRK3* affecting TGF-β signaling and TGF-β mediated cell behavior, we also assessed the TGF-β, BMP signaling and EMT markers in colon cancer cells after transfection with *NTRK3* [4], [5]. However, we did not observe a significant change in the expression of any of these proteins (**Figure S8**).

### Suppression of *NTRK3* induces transformed behavior in colon epithelial cells

**Figure 7:**
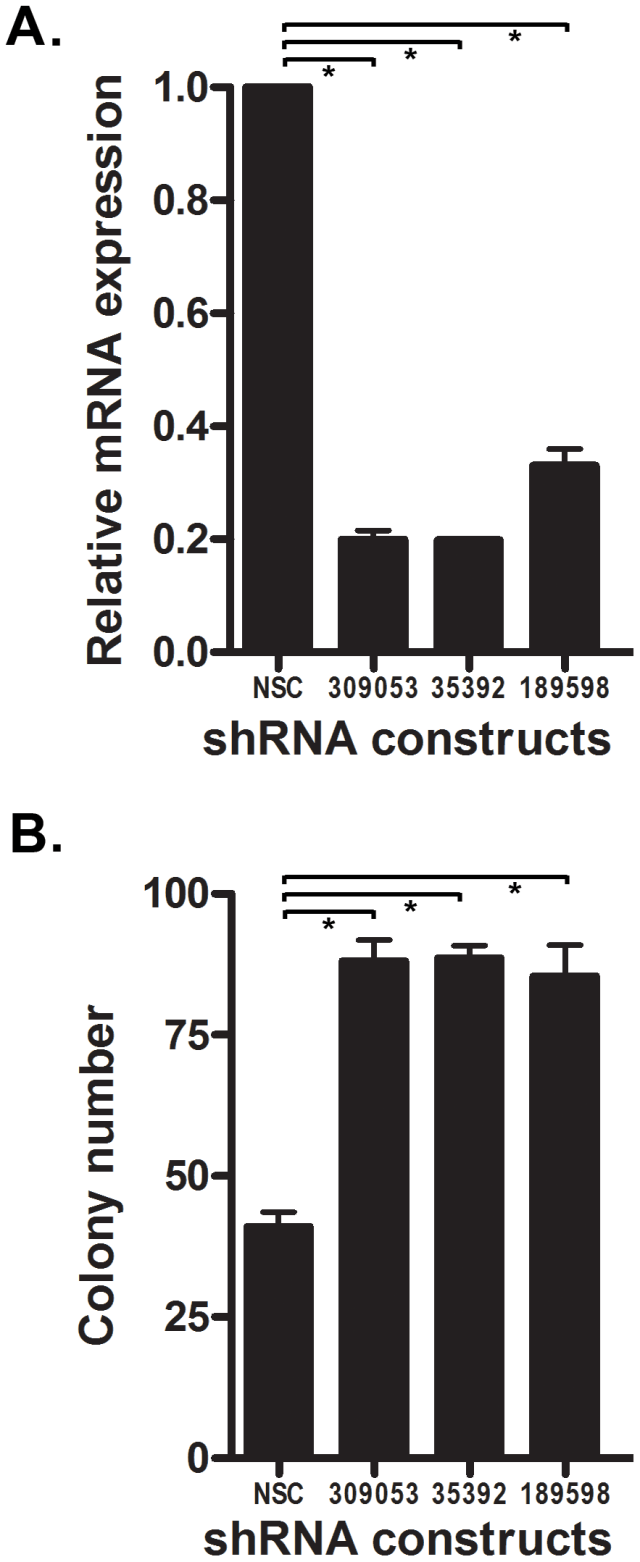
Knock-down of *Ntrk3* expression contributes to neoplastic transformation in normal colon epithelial cells. **A.** *Ntrk3* mRNA expression was efficiently knocked down by 3 different shRNA constructs (309053, 35392 and 189598) in the immortalized mouse colon epithelial cell line, YAMC. **B.** Suppression of *Ntrk3* promotes soft agar colony formation in the YAMC cells. The non-silencing control (NSC) is a control short-hairpin construct for the knockdown experiments. The asterisks indicate statistically significant differences. (*p*<0.05; two-sided student *t* test).

Since *NTRK3* is frequently methylated in colorectal adenomas, we carried out a series of studies to determine if loss of NTRK3 could induce transformed behavior in normal colon epithelial cells. We knocked down the expression of *Ntrk3* in an immortalized murine colon epithelial cell line (YAMC) and then assessed the cells for transformed behavior using a soft agar colony formation assay. As shown in **Figure 7**, the knockdown of *Ntrk3* was ∼80% as measured by RT-PCR, and this level of knockdown promoted anchorage independent growth in the YAMC cells. Of note, the parental YAMC cells grow slowly in soft agar. These findings suggest that loss of NTRK3 could be an early event in CRC formation.

### *NTRK3* suppresses the tumorigenic behavior of colon cancer cell lines both *in vitro* and *in vivo*

**Figure 8:**
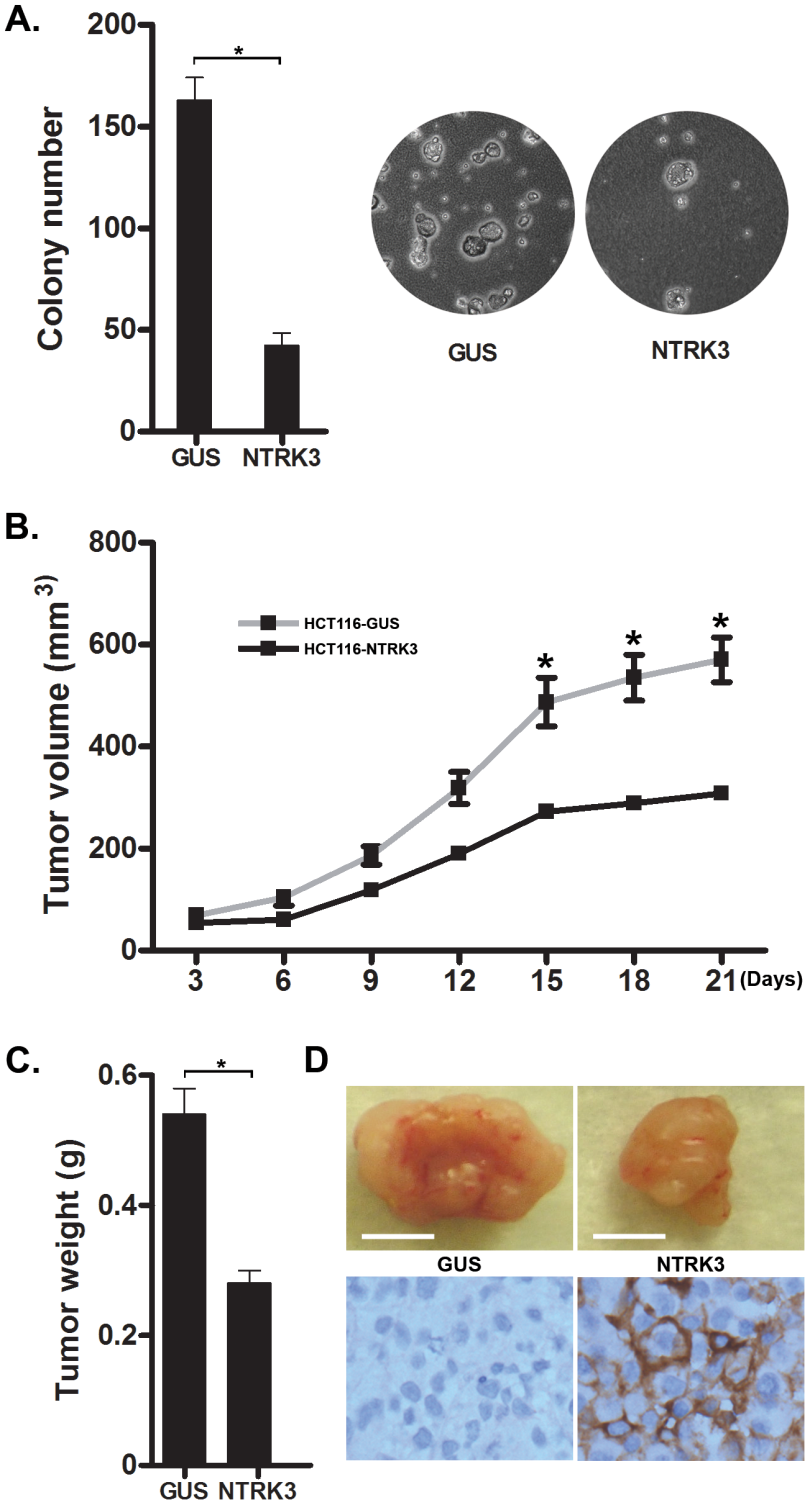
NTRK3 suppresses *in vitro* soft agar colony formation (A) and tumor xenograft growth (B–D) of colon cancer cell lines. **A.** The *GUS* and *NTRK3* stably transfected HCT116 cells were grown in soft agar for 2 weeks. Results are plotted as the mean colony numbers from three independent experiments. The asterisks indicate statistically significant differences (*p*<0.05; two-sided student *t* test). Representative fields depicting colonies of HCT116 cells grown in soft agar are shown. **B.** The growth of the HCT116-*NTRK3* and HCT116-*GUS* xenograft tumors in the *nu/nu* mice was measured over 3 weeks. The mean tumor volume is indicated, and the asterisks indicate statistically significant differences between the mean tumor volume of the *NTRK3*-reconstituted and control tumors (two-sided student *t* test). **C.** Tumors were removed 21 days after subcutaneous injection. The final tumor weight in NTRK3-expressing tumors was significantly less than the weight of the control tumors (*p* = 0.0021, n = 10). **D.** Representative tumors are shown (scale bar: 5 mm) along with confirmatory NTRK3 immunostaining.

We also performed studies on anchorage independent growth and tumor xenograft formation in established CRC cell lines to assess the putative tumor suppressor role of *NTRK3* in CRC. First, we assessed the effect of *NTRK3* expression on soft agar colony formation in HCT116 (MSI), RKO (CIMP) and HT29 (CIMP/MSS) cells. Transfection of full-length *NTRK3* induced a nearly 5- to 10-fold reduction in colony number of HCT116 (**Figure 8A**), RKO and HT29 cells (**Figure S9A and B**). We also assessed the tumor-suppressor activity of *NTRK3* in xenografts in immunodeficient *nu/nu* nude mice. *NTRK3* reconstitution significantly suppressed tumor growth compared to xenografts containing a control vector (**Figure 8B**). Twenty-one days after subcutaneous injection of the cells, the mice were sacrificed and the tumors were excised and measured. We found that both the size and weight of the NTRK3-expressing tumors were significantly reduced compared to the control xenografts (**Figure 8C** **and Figure S10**). NTRK3 expression in the xenografts from the cells transfected with *NTRK3* was confirmed by IHC (**Figure 8D**). Taken together, these results provide support for a tumor suppressor role for *NTRK3* in CRC.

## Discussion

The aberrant methylation of CpG islands in the promoter regions of genes is a common event in many cancers [19]. The average colon cancer genome contains 1,000–3,000 abnormally methylated genes [20]. In many cases, the aberrant methylation of these genes can silence the expression of tumor suppressor genes and consequently promote tumor formation. However, it is also apparent that the hypermethylation of many genes in cancer has no effect on the expression of the methylated gene and does not influence tumor formation. This later class of methylated genes is felt to represent passenger events in tumorigenesis [20]. Through a genome-wide screen for methylated genes in colon cancers, we identified methylated *NTRK3* in colon adenomas and adenocarcinomas. Methylated *NTRK3* was found in 67% of colorectal adenocarcinomas and 60% of adenomas. With regards to the functional significance of this epigenetic alteration, we found that the aberrant methylation of *NTRK3* suppressed *NTRK3* expression, which suggested *NTRK3* might act as a tumor suppressor gene in colon cancer. Our findings are in contrast to other studies in breast cancer that have demonstrated that *NTRK3* is oncogenic [4]. These opposing results appear to be a consequence of NTRK3 being a dependence receptor, which means that it can induce proliferation when it binds its ligand, NT-3, but induces apoptosis when NT-3 is not available [6]. Because NT-3 is expressed in the colon epithelium but not in colon neoplasms, our findings suggest that silencing of NTRK3 releases colon cancer cells from NTRK3-mediated apoptosis. These findings suggest that *NTRK3* might function as a novel conditional tumor suppressor gene in CRC.

Although somatic mutations of *NTRK3* that are predicted to inactivate function have been observed in CRC, *NTRK3*'s role as a tumor suppressor gene in CRC has not been clearly demonstrated to date [15]. In the present study, we have provided evidence that *NTRK3* can have conditional tumor suppressor activities in CRC. A similar role for *NTRK3* in neuroblastomas has recently been shown [6]. Reconstitution of *NTRK3* in the absence of NT-3, the ligand for NTRK3, induced caspase-related apoptosis and cell death in the colon cancer cell lines RKO, HT29 and HCT116. We found that the effects on apoptosis could be suppressed by the treatment of the NTRK3 expressing cell lines with NT-3. Perhaps most importantly, NTRK3 inhibited colony formation in soft agar colony formation assays and suppressed the growth of tumor xenografts, which are hallmark *in vitro* effects of tumor suppressor genes. In addition, we have shown that the naturally occurring *NTRK3-L760I* mutation impairs NTRK3's ability to induce apoptosis and suppress anchorage independent growth. These findings suggest that *NTRK3* is a CRC tumor suppressor gene that is inactivated by both genetic and epigenetic mechanisms.

The demonstration of *NTRK3* as a potential conditional tumor suppressor gene in the colon suggests NTRK3 may be the latest member of a class of dependence receptors that suppress colon cancer formation. Other conditional tumor suppressor genes identified in CRC and other cancers, include *DCC*, *UNC5C*, *p75^NTR^* and *MET* [12], [21]. The dependence receptor model purports that some receptors induce different biological effects on cells depending on whether they are in a ligand-bound or ligand-free state. These receptors can induce caspase-mediated apoptosis in the absence of ligand, but induce proliferation when bound by their ligands. Therefore, one of the critical aspects of this study is the assessment of the expression of the NTRK3 ligand NT-3 in the colon. NTRK3's preferred ligand, NT-3, was found to be substantially suppressed in both colorectal adenomas and adenocarcinomas, presumably secondary to hypermethylation of the *NT3* promoter region. It is plausible that the loss of NT-3 expression precedes the loss of NTRK3, which would create a clonal survival advantage for those CRC cells that silence *NTRK3*. Our studies suggest that inactivation of *NTRK3* occurs early in the polyp→cancer sequence and that it contributes to the transformation of colon epithelial cells.

With regards to the results of our studies, it is also important to consider the effects of loss of NT-3 and NTRK3 in the context of the entire neurotrophin receptor and ligand families because cross-talk between the ligand and receptor family members can occur. It has been shown that a precursor of NT-3, proNT-3, can activate p75^NTR^ and that NT-3 can activate NTRK1 or NTRK2, although this happens with low efficiency [18]. However, despite the potential for cross-talk, we did not observe any effects on colon cancer cells that lacked NTRK3 after being treated with NT-3. Therefore, our findings suggest that NTRK3 is the primary and perhaps only receptor for NT-3 in the colon and in colon neoplasms.

When bound to NT-3, NTRK3 functions as a typical receptor tyrosine kinase. Its activation is stimulated by neurotrophin-mediated dimerization and transphosphorylation of an activation loop tyrosine [22]. The major pathways activated by the NTRKs are MAPK, PI3K and PLC-γ1, among others [18], [22], [23]. Activation of the NTRKs and p75^NTR^ promote activation of NF-κB, and p75^NTR^ can activate the JNK pathway [18], [24], [25]. Previous studies have demonstrated that the activation of the MAPK and PI3K pathways by NTRK3 promotes cell differentiation, which in turn affects tumor progression [4], [23]. In this study, we also found that NTRK3 expression can activate the MAPK pathway. However, in this context the activation of ERK1/2 appears to be involved in the apoptotic response in colon cancer cells. There is a possibility that the MAPK activation we observed in this setting is an indirect effect of NTRK3 and a consequence of unopposed activation of p75^NTR^ [18]. Our studies do not allow us to exclude this possibility, although even if such a mechanism was present, it would not change the interpretation of *NTRK3* as being a colorectal cancer tumor suppressor gene.

In summary, we have identified *NTRK3* as a novel conditional tumor suppressor gene in the colon that is inactivated by epigenetic and genetic mechanisms. We have provided evidence that NTRK3 can trigger apoptosis and inhibit tumor growth in the absence of its ligand NT-3 and that these effects are reversed by the addition of NT-3. We also showed that suppression of NTRK3 can induce transformed behavior in immortalized colon epithelial cells. Our studies provide further insight into the complex relationship between NTRK3 and NT-3 in cancers as well as into dependence receptor biology in the colon. This class of tumor suppressor genes may offer new therapeutic strategies in the colon.

## Materials and Methods

### Cell lines, tissues and nucleic acid extraction

All studies in this manuscript have been approved by the FHCRC IRB committee and the IACUC committee. The studies of human tissues were all done on anonymous samples. The IRB protocol covering this study is IRB 1989 and is available upon request.

Nine human colorectal cancer cell lines (SW480, Vaco400, LS174T, HT29, Vaco576, RKO, Vaco503, HCT116 and Lovo) representing the spectrum of CRC molecular subtypes [MSI, CIN (aka MSS) and CIMP] were used. The cell lines were either purchased from ATCC or were kindly provided by Sanford Markowitz (Case Western Reserve University School of Medicine and Case Medical Center, Cleveland, OH). All cell lines had their identity confirmed by DNA genotyping. Some of the cell lines were treated with the DNMT1 inhibitor (5 µM) 5-aza-2′-deoxycytidine (5-AZA; Sigma) in the experiments in this study.

Primary tissue samples used in the methylation array studies (N = 8 CRC's) were obtained from the ColoCare CRC cohort study (Fred Hutchinson Cancer Research Center, Seattle, WA) and from healthy individuals undergoing screening colonoscopy at the University of Washington Medical Center (Seattle, WA) (N = 6). Detailed information on these samples is shown in **Table S2**.

Formalin-fixed, paraffin-embedded (FFPE) and fresh-frozen colon neoplasms and normal colon tissue samples were obtained from the pathology archives at Vanderbilt University Medical Center (Nashville, TN), the Department of Veterans Affairs Tennessee Valley Health Care System, Meharry Medical Center (Nashville, TN), and the University Hospital of Cleveland (Cleveland, OH) following IRB approved protocols at each institution. Colorectal cancers, colon adenomas, and adjacent normal tissue samples were also provided by the Cooperative Human Tissue Network. In total, these samples included 52 cases of histologically normal colonic mucosa from individuals without cancer or inflammatory bowel disease (IBD) and 25 samples of histological normal colonic mucosa from individuals who had undergone colon resection for CRC or colon adenomas.

DNA and RNA were extracted from these samples as previously described [26].

### Methylation array studies

These studies were conducted using Infinium HumanMethylation450 BeadChip arrays (Illumina) with DNA from CRCs (N = 8) and normal colon epithelium samples from cancer-free individuals (N = 6). Specific details regarding the platform, sample preparation and data filtering strategies have been described in our previous studies [3].

### Sodium bisulfite conversion of genomic DNA

Bisulfite conversion of DNA was performed as described previously [27], [28].

### Bisulfite sequencing

For sequencing, bisulfite-converted DNA was PCR amplified, and the amplicons were then subjected to direct sequencing. The primers are described in detail in **Table S3**. The sequencing was conducted as described previously [27].

### Quantitative methylation-specific PCR

Quantitative methylation-specific PCR (qMSP; MethyLight) was performed using an ABI Prism 7700 detection system (Applied Biosystems). Detailed methods are provided in the **Text S1** as well as in previous publications [27]. The primers and probes targeting *NTRK3* are described in detail in **Table S3**.

### Molecular characterization of colorectal neoplasms

The CpG Island Methylator Phenotype (CIMP) status and Microsatellite instability (MSI) status of a subset of the colorectal neoplasms were determined as described previously [27]. The gene mutation status of *KRAS*, *BRAF*, *APC*, *TP53* and *PIK3CA* was assessed by using the qBiomarker Somatic Mutation PCR System Arrays/Human Colon Cancer (Qiagen) following the manufacturer's protocol.

### Cell culture, plasmid constructs and transfection

Human CRC cell lines (SW480, Vaco400, LS174T, HT29, Vaco576, RKO, Vaco503, HCT116, and Lovo) were grown in Dulbecco's Modified Eagle media (DMEM; Invitrogen) supplemented with 10% fetal bovine serum (FBS; Invitrogen). The YAMC (Young Adult Mouse Colon) cell line was a kind gift from Dr. Robert H. Whitehead (Vanderbilt University School of Medicine, Nashville, TN) and was cultured as described previously [29]. To investigate the effects of re-expression of *NTRK3*, HCT116 and RKO cells were incubated for 72 hours with 5 µM 5′-AZA (Sigma). The media was replaced every 24 hours with fresh 5′-AZA. After 72 hours of treatment, the cells were washed twice with PBS and then grown in drug-free media for another 72 hours before harvesting.

The full-length *NTRK3* cDNA (IOH54159, Invitrogen) was subcloned into the pDEST27 Vector (Invitrogen) to create pDEST27-*NTRK3*. The correct orientation of the insert was confirmed by restriction digest.

The pDEST27-based plasmids containing *NTRK3-G608S*, *NTRK3-I695V* and *NTRK3-L760I* were constructed based on the wild-type *NTRK3* expression vector by using the GENEART site-direct mutagenesis system (Invitrogen) following the manufacturer's protocol. Successful mutagenesis was confirmed by direct sequencing (**Figure S5**). The sequencing primers are described in **Table S3**.

pGIPz-based short hairpin RNA (shRNA) constructs specifically targeting mouse *Ntrk3* mRNA were purchased from Thermo Scientific/Open Biosystems Mouse shRNAmir Libraries maintained by the Genomics Shared Resource Core at the Fred Hutchinson Research Center (FHCRC, Seattle, WA). The details regarding the target regions of each shRNA are shown in **Table S3**. All 3 shRNA constructs were confirmed by direct sequencing (**Table S3**). Lentivirus containing the shRNA constructs were generated by co-transfecting pGIPz-shRNA with the packaging plasmid psPAX2 and the envelope plasmid pMD2.G (kindly provided by Michael Davis, FHCRC, Seattle, WA) into 293T packaging cells. Virus supernatant was filtered through a 0.2-µm filter and stored at −80°C until use. shRNA-mediated knock-down studies were performed by infecting YAMC cells with shRNA lentivirus for 48 hours, followed by an additional 72 hours of puromycin selection (4 µg/mL; Invitrogen). The stable YAMC cells were maintained in media containing 2 µg/mL puromycin. pGIPz-shNSC (Thermo Scientific/Open Biosystems), which expresses a scrambled shRNA with no known target sequence in the mouse genome, was used as a control for the transduction procedure.

HCT116 (MSI), RKO (CIMP) and HT29 (CIMP/MSS) cells were transiently transfected with pDEST27-based constructs (Invitrogen) using the XtremeGENE 9 DNA transfection reagent (Roche) following the manufacturer's protocol. Transfected cells were grown for 10–14 days in media containing 2400 µg/mL G418 (RKO cells) (Invitrogen) or 1200 µg/mL G418 (HCT116 and HT29 cells). For the experiments using NT-3, recombinant human neurotrophin-3 (NT-3) (R&D System) was added into the culture media 24 hours after transfection and incubated for another 24 hours.

### Quantitative RT-PCR

Total RNA was isolated from CRC cell lines and primary tissues using TRIzol (Ambion) and was purified with the RNeasy Mini kit (Qiagen) according to the manufacturers' protocols. For human samples, TaqMan On-Demand primers and probes (Applied Biosystems) were used to determine the relative expression levels of *NTRK3* (Hs00176797_m1) and *NT-3* (Hs01548350_m1). *GUSB* (Hs99999908_m1) was used as a RNA input loading control. For YAMC cells (mouse) related RT-PCR, *Ntrk3* (Mm00456222_m1) and *Gusb* (Mm01197698_m1) were used. All reactions were run in triplicate on an ABI Prism 7700 detection system.

### Immunohistochemistry

FFPE sections of CRCs, adenomas and matched normal colonic mucosa were subjected to immunostaining with a rabbit anti-human TrkC monoclonal antibody (C44h5, Cell Signaling Technology). Briefly, 4 µm tissue sections were deparaffinized, rehydrated, and subjected to antigen retrieval by boiling in sodium citrate buffer (10 mmol/L, pH 6.0). The sections were incubated for 60 minutes with TrkC primary antibody (1∶50), stained with 3,3-diaminobenzidine, counterstained with hematoxylin, and mounted as described previously [30].

### *In vitro* apoptosis assays

Programmed cell death was analyzed using the Cell Death Detection ELISA (Roche Molecular Biochemicals) following the manufacturer's instructions. Briefly, 48 hours after transfection or 24 hours after treatment with NT-3 or a selective ERK inhibitor, the cells were lysed, and the lysates analyzed using the ELISA kit.

Caspase-3 and caspase-7 activity was measured using the Caspase-Glo3/7 Assay (Promega) following the manufacturer's protocol. Relative caspase activation was calculated as the ratio of the caspase activity of the *NTRK3-* transfected cells and the negative control *GUS*-transfected cells.

### Soft agar colony formation assay

The soft agar colony formation assays were performed as previously described [26], [27]. Briefly, 6,000 stably transfected HCT116, RKO or YAMC cells were mixed with 0.4% Sea Plaque agarose containing tissue culture media (top layer) and pipetted onto a solidified layer of 0.8% Sea Plaque agarose containing tissue culture media (bottom layer) in 35 mm petri dishes in triplicate. The tissue culture media was exchanged every 2∼3 days. After 14 days, colonies larger than 50 µm in diameter were counted using a phase-contrast microscope equipped with a reticule in 4 randomly selected fields in the three replicate dishes. The experiment was performed in triplicate.

### Western immunoblotting

Western blotting experiments were conducted as described previously [30]. Briefly, cells were lysed using RIPA buffer containing Phosphatase Inhibitor Cocktail (P5726, Sigma) at 0°C. The protein extracts (50 µg/sample) were subjected to electrophoresis through 12% Bis-Trispolyacrylamide gels (BioRad) and then transferred to PVDF membranes. Antibodies used were purchased from Cell Signaling Technology: anti-phospho-Akt (Ser473, #9271), anti-phospho-NF-κB (Ser536, #3033), anti-phospho-Erk1/2 (Thr202/Tyr204, #4370), anti-phospho-Smad1 (Ser463/465)/Smad5 (Ser463/465)/Smad8 (Ser426/428) (#9511), anti-phospho-Smad2 (Ser465/467, #3101), anti-phospho-Smad3 (C25A9, Ser423/425, #9520), anti-Smad2/3 (#3102), anti-TrkC (C44h5, #3376), anti-BMPR2 (#6979), and Epithelial-Mesenchymal Transition (EMT) antibodies, anti-N-Cadherin (#4061), anti-Vimentin (D21H3, #5741), anti-E-Cadherin (24E10, #3195), anti-ZO-1 (D7D12, #8193), anti-Snail (C15D3, #3879). Immunoreactive proteins were then visualized by incubating the PVDF membranes with ECL plus detection reagents, followed by imaging of chemiluminescence on an imager (X-ray film-based).

### Tumor xenograft studies

The care and use of the mice was conducted following protocols approved by the Fred Hutchinson Cancer Research Center IACUC. The IACUC protocol covering this work is IACUC 1624 and is available upon request. All IACUC protocols at the FHCRC require that animal suffering is eliminated unless required for the studies, in which case extensive justification is required.

Three to four week-old female athymic *nu/nu* mice were obtained from Harlan Laboratories. The mice were housed for one week in a pathogen-free animal facility prior to tumor cell injection. 1×10^7^ *GUS* or *NTRK3* stably transfected HCT116 cells in 200 µL DMEM and Matrigel (1∶1 mix) (BD Biosciences) were injected subcutaneously into the right flank of each mouse. The tumor sizes were measured using a caliper, and the tumor volume was calculated as follows: 0.5(length×width^2^) [6]. The mice were assessed every three days and sacrificed at three weeks after injection.

### Statistical analyses

Receiver operating characteristic (ROC) curves and area under the curve (AUC) for *NTRK3* methylation frequency of primary tissues were constructed on the basis of methylation levels. The Chi-squared test was used to compare the frequency of methylated *NTRK3* between cancer and normal samples. The Fisher's exact test was used to test the association between the *NTRK3* methylation status and clinical/molecular characteristics of CRC patients. Student *t* test or analysis of variance (ANOVA) was used to analyze the RT-PCR data. The Mann-Whitney rank sum test was used to analyze the data obtained from the cell death and apoptosis assays. Statistical analysis was performed using SPSS 13.0 software. All *p* values are two-sided, and a *p* value<0.05 was considered statistically significant.

## Supporting Information

Figure S1 Methylated *NTRK3* occurs independently of other methylated genes that commonly occur in CRC. A heat map that shows the relative methylation levels of *NTRK3*, *MLH1*, *CDKN2A/p16*, and *RASSF1A* genes in the 8 CRC cases run on the HumanMethylation450 arrays is displayed. There is no significant correlation between methylated *NTRK3* and methylation of any of these genes. The methylation levels are quantified as follows: “1” stands for 100% methylated; “0” represents 100% unmethylated DNA. The red color represents higher methylation levels (towards “1”) whereas green represents lower methylation levels (towards “0”).
(TIF)

Figure S2 *NT3* mRNA expression is suppressed by methylation in colorectal cancer cell lines. **A.** There is no *NT3* expression in RKO, HCT116, FET, Vaco400 and HT-29 cells. Low-level *NT3* expression is apparent in SW480, Lovo, LS174T, and AAC1/SB10. The expression level is substantially less than that observed in the normal colon, which was on average 3.8±0.66 (**Figure 3**). **B.** *NT3* mRNA expression in HCT116 and RKO cells is induced by treatment with the DNMT1 inhibitor 5-aza-2′deoxycytidine (5-AZA) when compared to treatment with the vehicle alone (“Mock”). **C.** *NT3* methylation status of colorectal cancer cell lines as determined by methylation specific PCR (qMSP) demonstrates that cells with no *NT3* mRNA expression carry methylated *NT3*, whereas cell lines that express *NT3* have unmethylated *NT3*. These levels of expression are very low. (Methyl: universal methylated control DNA; Unmethyl: universal unmethylated control DNA; H_2_O: no template control). M = methylated, U = unmethylated.
(TIF)

Figure S3 *NTRK3* mRNA and protein expression induced by *NTRK3*-transfection of cell lines that carry methylated *NTRK3*. HCT116, RKO and HT29 cells carry methylated *NTRK3* and have no detectable *NTRK3* mRNA expression (**A**) or protein expression (**B**) when transfected with the mock conditions (Mock) or GUS control vector (GUS). *NTRK3* mRNA and protein expression can be detected in all three cell lines after transfection with the vector expressing *NTRK3* (NTRK3). The bands seen in the western blot are the expected sizes for NTRK3 (100 kd and 145 kd).
(TIF)

Figure S4 Assessment of apoptosis after reconstitution of *NTRK3* in HCT116 (**A**), RKO (**B**) and HT29 (**C**). NTRK3 induces cell death in HCT116 (MSI), RKO (CIMP) and HT29 (MSS) cells, and NT-3 (100 ng/mL) inhibits this effect in all three cell lines. DMSO treatment was used as a control for nonspecific effects. HCT116, RKO and HT29 carry methylated *NTRK3*. The asterisks indicate statistically significant differences, *p*<0.05 as determined by a 2-sided Mann-Whitney rank sum test. All values were normalized to the GUS transfected, DMSO treated cells.
(TIF)

Figure S5 Direct sequencing results of mutant *NTRK3* constructs (based on somatic mutations G608S, I695V, and L760I found in human colon cancer samples). The arrow indicates the mutated basepair and confirms the success of the site-directed mutagenesis used to generate the mutant. **A.** Sequencing results for *NTRK3-G608S* mutant. **B.** Sequencing results for *NTRK3-I695V* mutant. **C.** Sequencing results for *NTRK3-L760I* mutant.
(TIF)

Figure S6 Inhibition of MAPK activity after *NTRK3* reconstitution in RKO cells decreases NTRK3 induced apoptosis. Caspase activity in the CRC cell line RKO was quantified 48 hours after transfection with NTRK3 using the Caspase-Glo 3/7 assay. The selective MAPK/ERK pathway inhibitor, U0126 (10 µM), significantly decreases caspase activity in the RKO cell line transfected with *NTRK3*. 16% FBS (30 minute treatment) was used to stimulate the cells before protein harvest as a positive control for ERK activation. All experiments were performed in triplicate, and the results shown are fold changes compared to the empty vector control. The asterisks indicate statistically significant differences, p<0.05 as determined by a 2-sided Mann-Whitney rank sum test.
(TIF)

Figure S7 NT-3 suppresses the expression of reconstituted *NTRK3* and suppresses ERK1/2 phosphorylation in the colon cancer cell line HCT116. HCT116 cells were transfected with either *GUS* or *NTRK3* followed by treatment with NT-3 (100 ng/mL for 24 hours) 24 hours after transfection. The phosphorylation of Erk1/2 induced by NTRK3 expression is effectively blocked by NT-3, presumably in part through suppression of NTRK3 protein expression.
(TIF)

Figure S8 *NTRK3* transfection does not alter the TGF-β, BMP signaling pathways or contribute to epithelial-mesenchymal transition (EMT) in the colorectal cancer cell line RKO treated with or without NT-3. RKO cells were transfected with either *GUS* or *NTRK3* as indicated. Protein lysates were collected after NT-3 (100 ng/mL) treatment for 24 hours. TGF-β (represented by p-Smad2/3) and BMP (represented by p-Smad1/5/8 and BMPR2) signaling pathways were not affected by reconstitution of NTRK3. None of the EMT markers (N-Cadherin, Vimentin, E-Cadherin, ZO-1 and Snail) was significantly altered by tranfection with *NTRK3* regardless of NT-3 treatment. Actin was used as a loading control.
(TIF)

Figure S9 NTRK3 suppresses *in vitro* soft agar colony formation in RKO (**A**) and HT29 (**B**) cells. The *GUS* and *NTRK3* stably transfected RKO (A) and HT29 (B) cells were grown in soft agar for 2 weeks. Results are plotted as the mean colony numbers for three independent experiments. The asterisks indicate statistically significant differences. (*p*<0.05; two-sided student *t* test).
(TIF)

Figure S10 Representative tumor xenografts dissected from *nu/nu* mice injected with HCT116 colon cancer cells transfected with *NTRK3* or the control vector (GUS). The *NTRK3* transfected tumors are smaller and appear less vascular when compared to the control tumor xenografts.
(TIF)

Figure S11 ROC analysis to determine the optimal percentage of methylated reference (PMR) for methylated *NTRK3* that detects cancer specific levels of *NTRK3* methylation. An ROC curve was constructed by plotting sensitivity vs. 1-specificity comparing adenocarcinomas (n = 76) to normal colon samples (n = 98). Area under the curve (AUC) for the sample set was 0.843 (95% CI0.781–0.904).
(TIF)

Table S1 Predicted effect of naturally occurring somatic mutations of *NTRK3* in colorectal cancer.
(DOCX)

Table S2 Clinical characteristics of the samples analyzed with the HumanMethylation450 array.
(DOCX)

Table S3 Primer and Probe Sequences for *NTRK3* reagents.
(DOCX)

Table S4 Results of ROC analyses of the methylation levels in colon adenocarcinomas detected by MethyLight.
(DOCX)

Text S1 Supplemental Methods. Description of methods used for studies whose data resulted in supplemental data and additional detailed description of methods used for studies in main text of manuscript.
(DOCX)
