# Supplementary figures and images for: NTRK3 Is a Potential Tumor Suppressor Gene Commonly Inactivated by Epigenetic Mechanisms in Colorectal Cancer
Source: PLoS Genet. 2013 Jul 11;9(7):e1003552. doi: 10.1371/journal.pgen.1003552 (PMC3708790; doi:10.1371/journal.pgen.1003552)

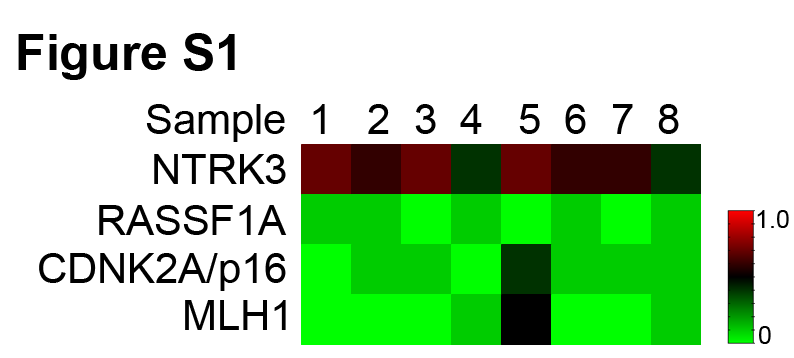

Supplement: Figure S1 — Methylated NTRK3 occurs independently of other methylated genes that commonly occur in CRC. A heat map that shows the relative methylation levels of NTRK3, MLH1, CDKN2A/p16, and RASSF1A genes in the 8 CRC cases run on the HumanMethylation450 arrays is displayed. There is no significant correlation between methylated NTRK3 and methylation of any of these genes. The methylation levels are quantified as follows: “1” stands for 100% methylated; “0” represents 100% unmethylated DNA. The red color represents higher methylation levels (towards “1”) whereas green represents lower methylation levels (towards “0”). (TIF) [file pgen.1003552.s001.tif]

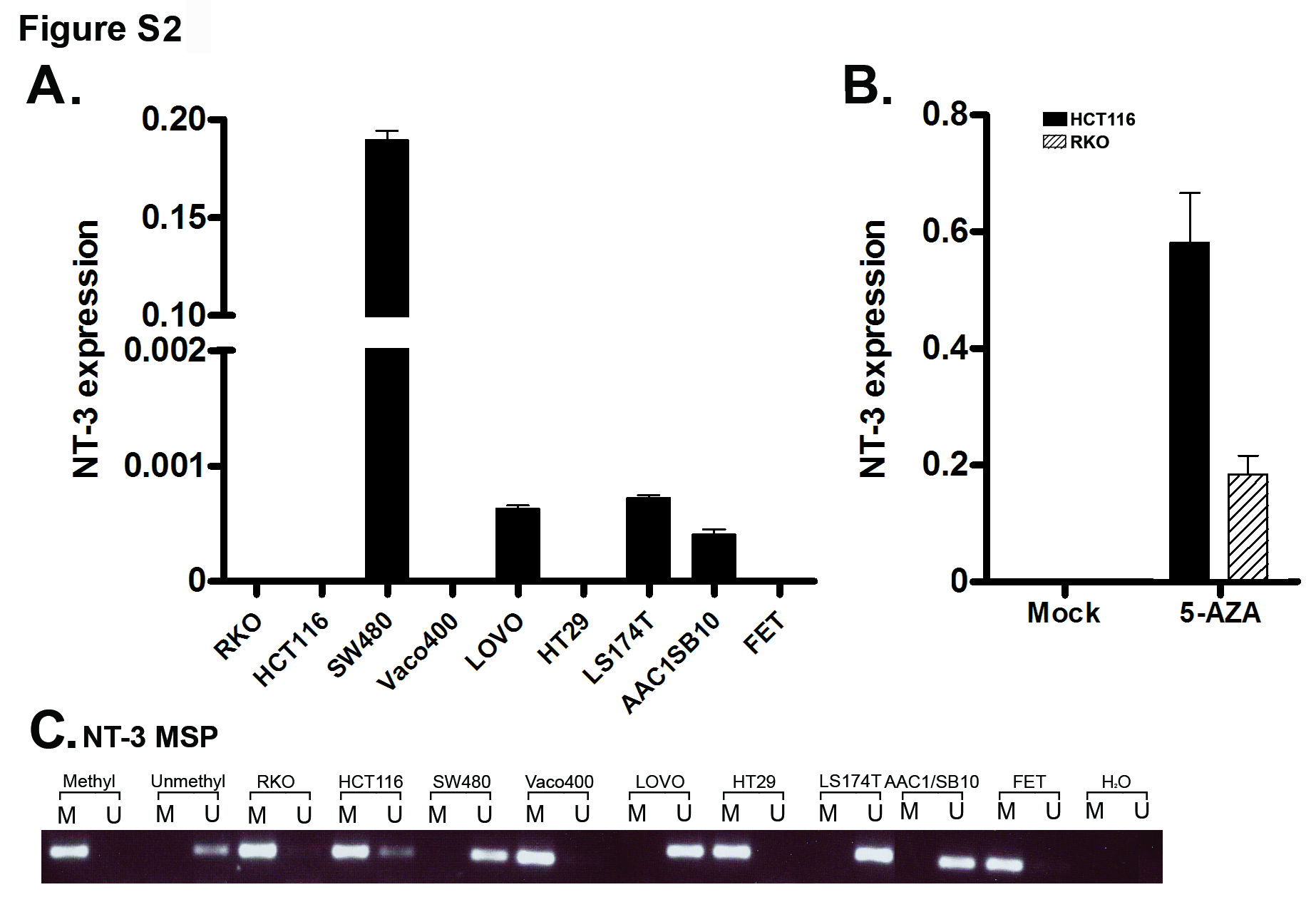

Supplement: Figure S2 — NT3 mRNA expression is suppressed by methylation in colorectal cancer cell lines. A. There is no NT3 expression in RKO, HCT116, FET, Vaco400 and HT-29 cells. Low-level NT3 expression is apparent in SW480, Lovo, LS174T, and AAC1/SB10. The expression level is substantially less than that observed in the normal colon, which was on average 3.8±0.66 ( Figure 3 ). B. NT3 mRNA expression in HCT116 and RKO cells is induced by treatment with the DNMT1 inhibitor 5-aza-2′deoxycytidine (5-AZA) when compared to treatment with the vehicle alone (“Mock”). C. NT3 methylation status of colorectal cancer cell lines as determined by methylation specific PCR (qMSP) demonstrates that cells with no NT3 mRNA expression carry methylated NT3, whereas cell lines that express NT3 have unmethylated NT3. These levels of expression are very low. (Methyl: universal methylated control DNA; Unmethyl: universal unmethylated control DNA; H2O: no template control). M = methylated, U = unmethylated. (TIF) [file pgen.1003552.s002.tif]

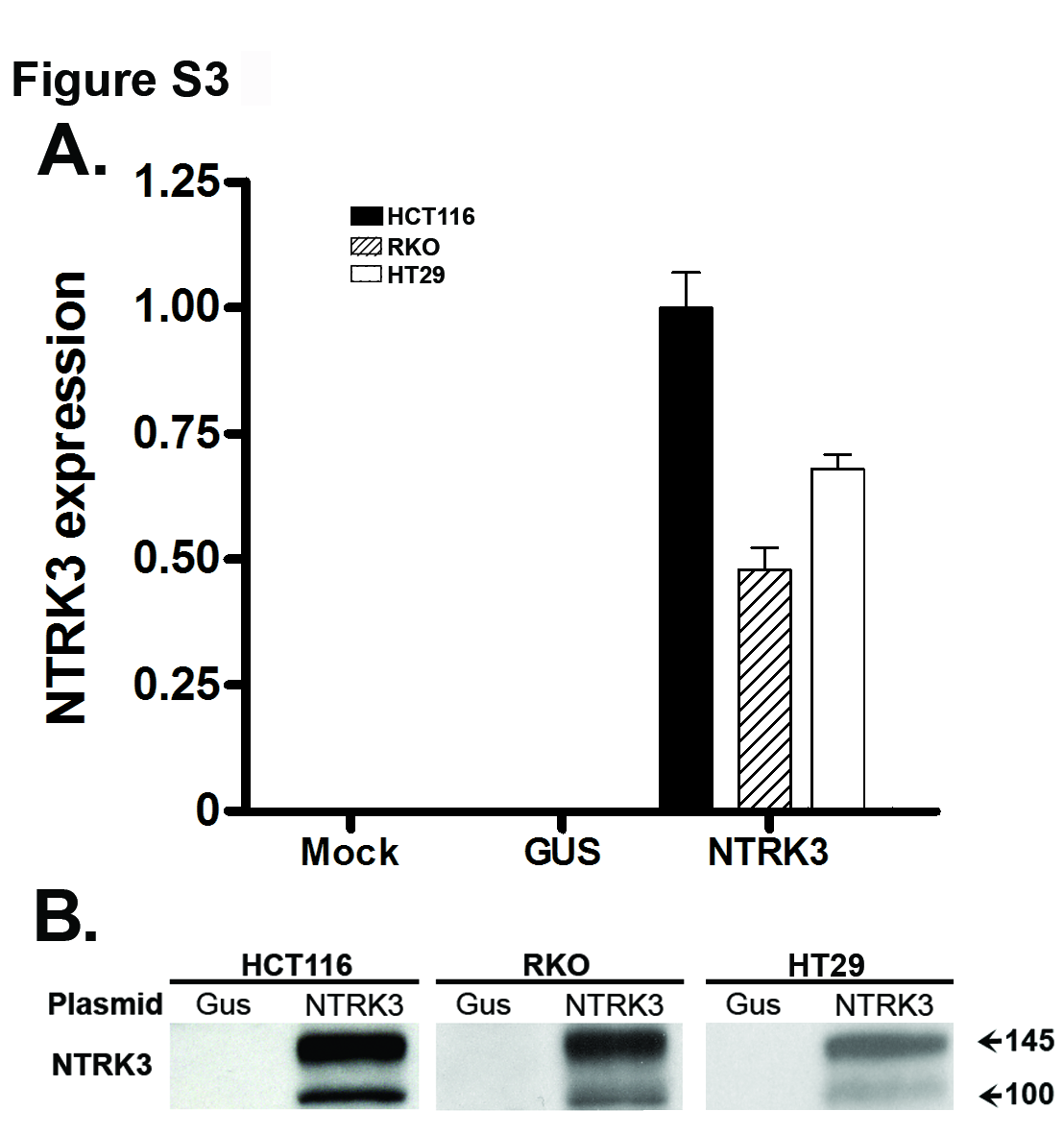

Supplement: Figure S3 — NTRK3 mRNA and protein expression induced by NTRK3-transfection of cell lines that carry methylated NTRK3. HCT116, RKO and HT29 cells carry methylated NTRK3 and have no detectable NTRK3 mRNA expression (A) or protein expression (B) when transfected with the mock conditions (Mock) or GUS control vector (GUS). NTRK3 mRNA and protein expression can be detected in all three cell lines after transfection with the vector expressing NTRK3 (NTRK3). The bands seen in the western blot are the expected sizes for NTRK3 (100 kd and 145 kd). (TIF) [file pgen.1003552.s003.tif]

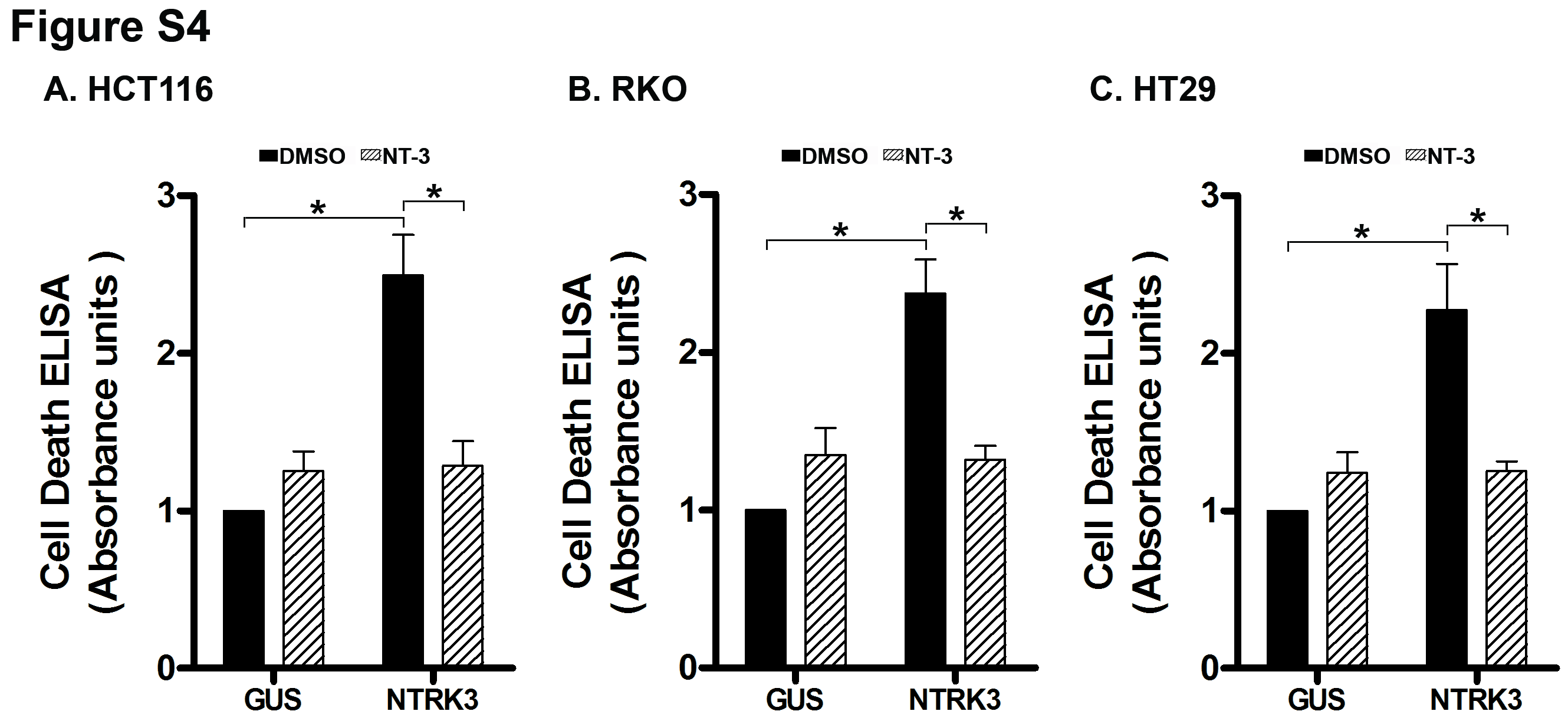

Supplement: Figure S4 — Assessment of apoptosis after reconstitution of NTRK3 in HCT116 (A), RKO (B) and HT29 (C). NTRK3 induces cell death in HCT116 (MSI), RKO (CIMP) and HT29 (MSS) cells, and NT-3 (100 ng/mL) inhibits this effect in all three cell lines. DMSO treatment was used as a control for nonspecific effects. HCT116, RKO and HT29 carry methylated NTRK3. The asterisks indicate statistically significant differences, p<0.05 as determined by a 2-sided Mann-Whitney rank sum test. All values were normalized to the GUS transfected, DMSO treated cells. (TIF) [file pgen.1003552.s004.tif]

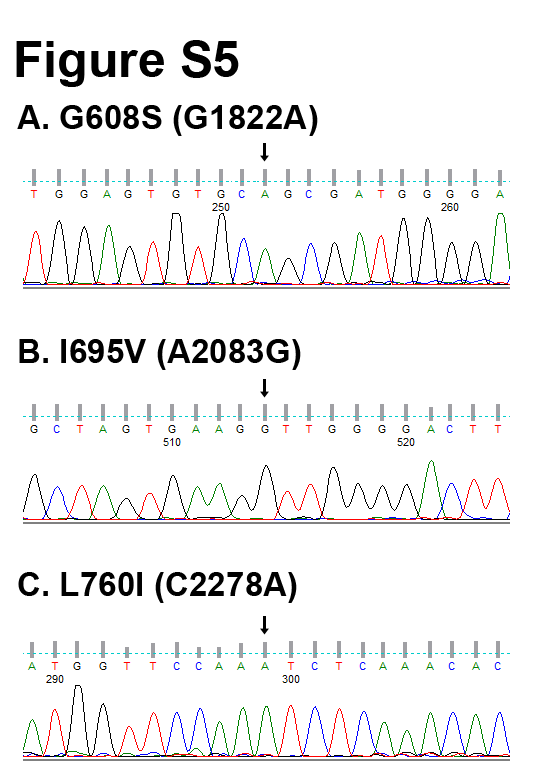

Supplement: Figure S5 — Direct sequencing results of mutant NTRK3 constructs (based on somatic mutations G608S, I695V, and L760I found in human colon cancer samples). The arrow indicates the mutated basepair and confirms the success of the site-directed mutagenesis used to generate the mutant. A. Sequencing results for NTRK3-G608S mutant. B. Sequencing results for NTRK3-I695V mutant. C. Sequencing results for NTRK3-L760I mutant. (TIF) [file pgen.1003552.s005.tif]

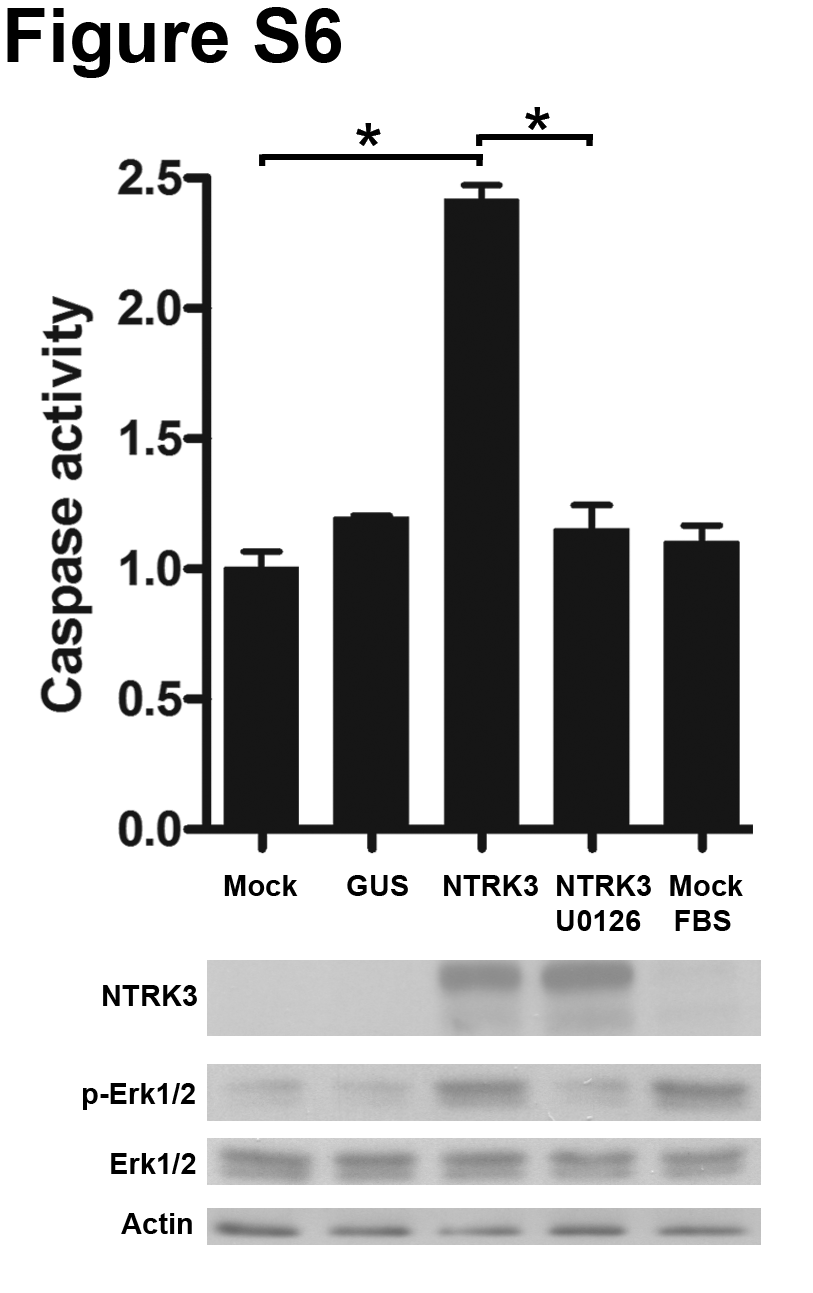

Supplement: Figure S6 — Inhibition of MAPK activity after NTRK3 reconstitution in RKO cells decreases NTRK3 induced apoptosis. Caspase activity in the CRC cell line RKO was quantified 48 hours after transfection with NTRK3 using the Caspase-Glo 3/7 assay. The selective MAPK/ERK pathway inhibitor, U0126 (10 µM), significantly decreases caspase activity in the RKO cell line transfected with NTRK3. 16% FBS (30 minute treatment) was used to stimulate the cells before protein harvest as a positive control for ERK activation. All experiments were performed in triplicate, and the results shown are fold changes compared to the empty vector control. The asterisks indicate statistically significant differences, p<0.05 as determined by a 2-sided Mann-Whitney rank sum test. (TIF) [file pgen.1003552.s006.tif]

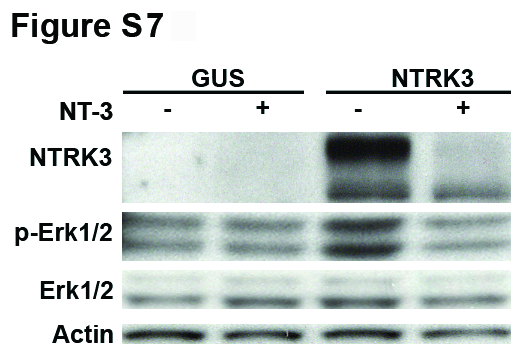

Supplement: Figure S7 — NT-3 suppresses the expression of reconstituted NTRK3 and suppresses ERK1/2 phosphorylation in the colon cancer cell line HCT116. HCT116 cells were transfected with either GUS or NTRK3 followed by treatment with NT-3 (100 ng/mL for 24 hours) 24 hours after transfection. The phosphorylation of Erk1/2 induced by NTRK3 expression is effectively blocked by NT-3, presumably in part through suppression of NTRK3 protein expression. (TIF) [file pgen.1003552.s007.tif]

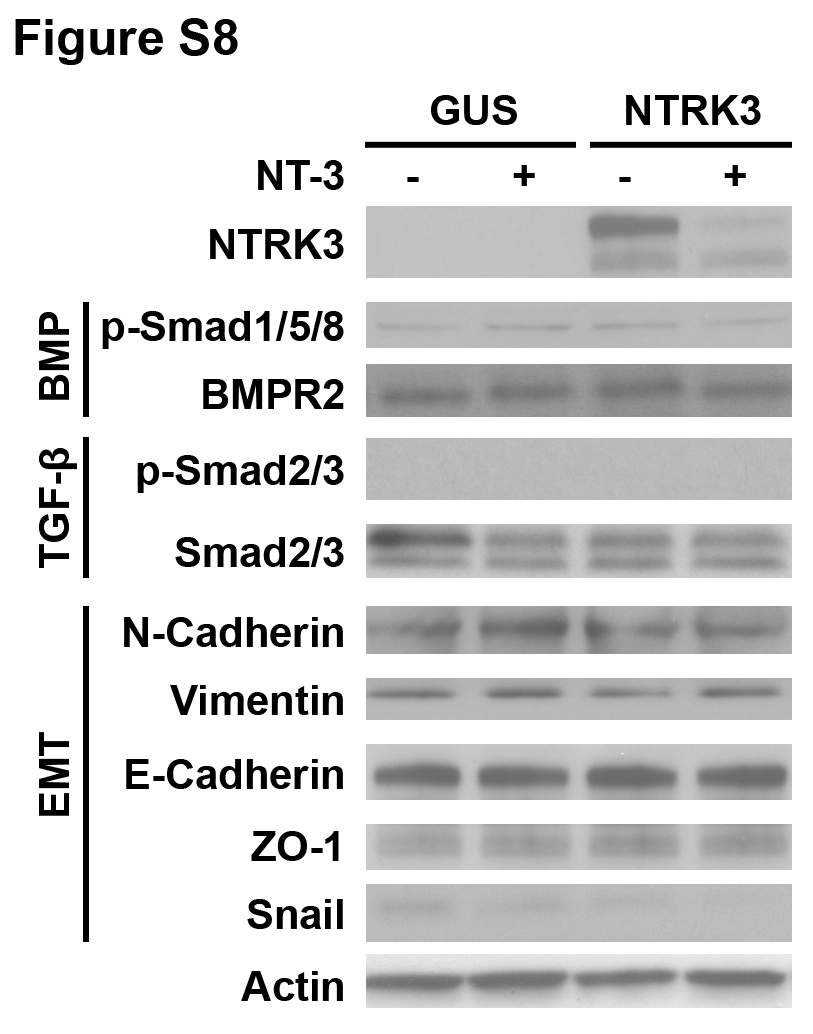

Supplement: Figure S8 — NTRK3 transfection does not alter the TGF-β, BMP signaling pathways or contribute to epithelial-mesenchymal transition (EMT) in the colorectal cancer cell line RKO treated with or without NT-3. RKO cells were transfected with either GUS or NTRK3 as indicated. Protein lysates were collected after NT-3 (100 ng/mL) treatment for 24 hours. TGF-β (represented by p-Smad2/3) and BMP (represented by p-Smad1/5/8 and BMPR2) signaling pathways were not affected by reconstitution of NTRK3. None of the EMT markers (N-Cadherin, Vimentin, E-Cadherin, ZO-1 and Snail) was significantly altered by tranfection with NTRK3 regardless of NT-3 treatment. Actin was used as a loading control. (TIF) [file pgen.1003552.s008.tif]

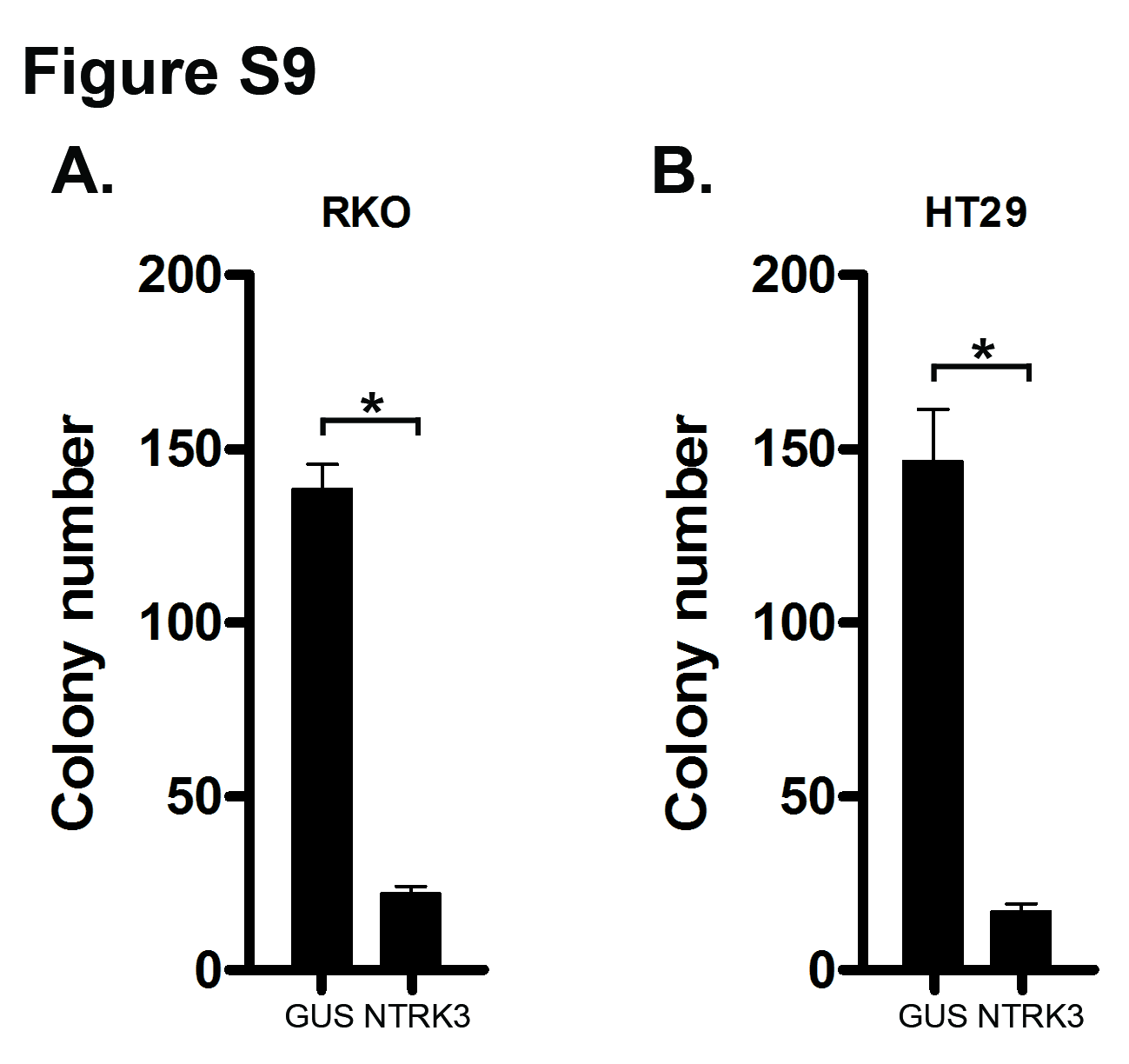

Supplement: Figure S9 — NTRK3 suppresses in vitro soft agar colony formation in RKO (A) and HT29 (B) cells. The GUS and NTRK3 stably transfected RKO (A) and HT29 (B) cells were grown in soft agar for 2 weeks. Results are plotted as the mean colony numbers for three independent experiments. The asterisks indicate statistically significant differences. (p<0.05; two-sided student t test). (TIF) [file pgen.1003552.s009.tif]

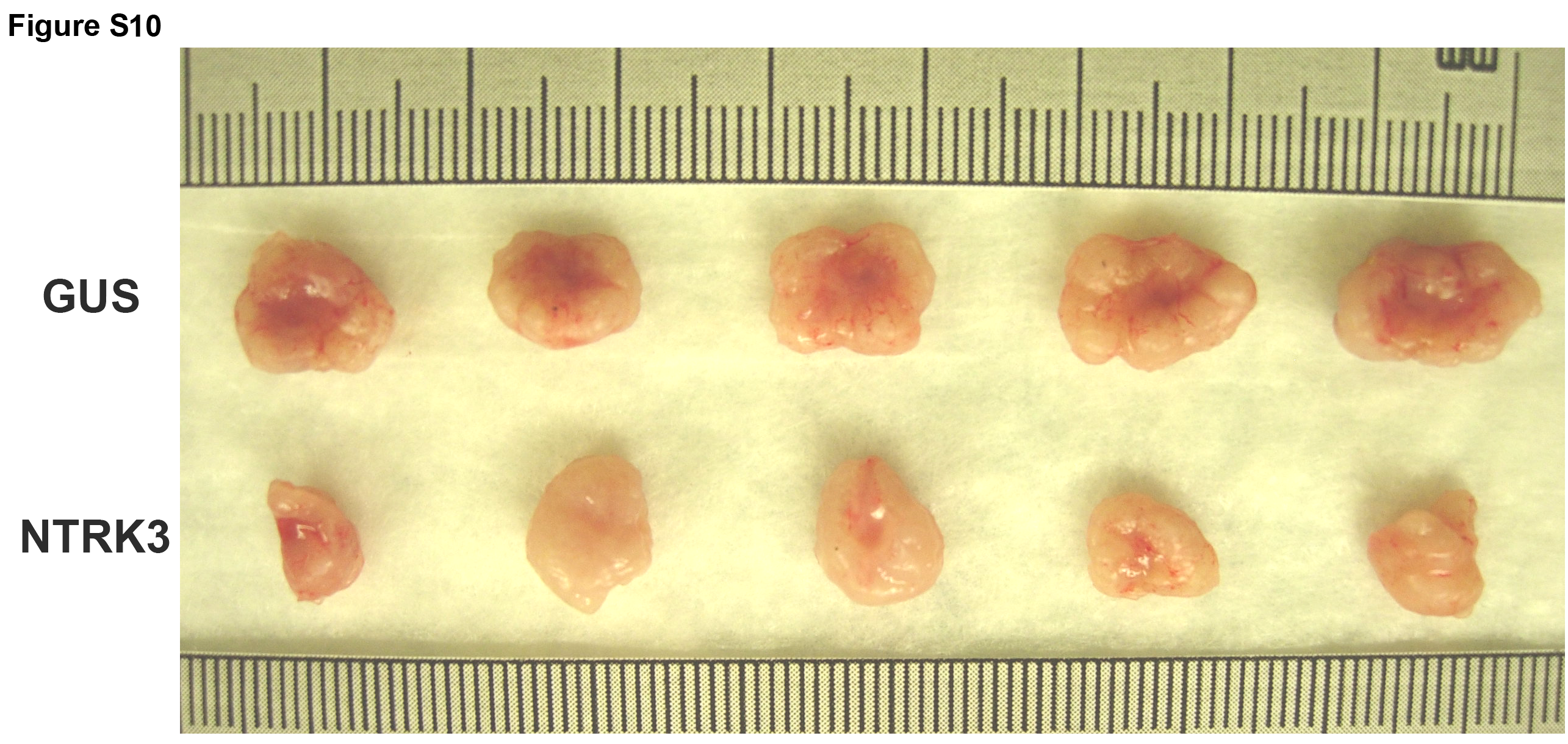

Supplement: Figure S10 — Representative tumor xenografts dissected from nu/nu mice injected with HCT116 colon cancer cells transfected with NTRK3 or the control vector (GUS). The NTRK3 transfected tumors are smaller and appear less vascular when compared to the control tumor xenografts. (TIF) [file pgen.1003552.s010.tif]

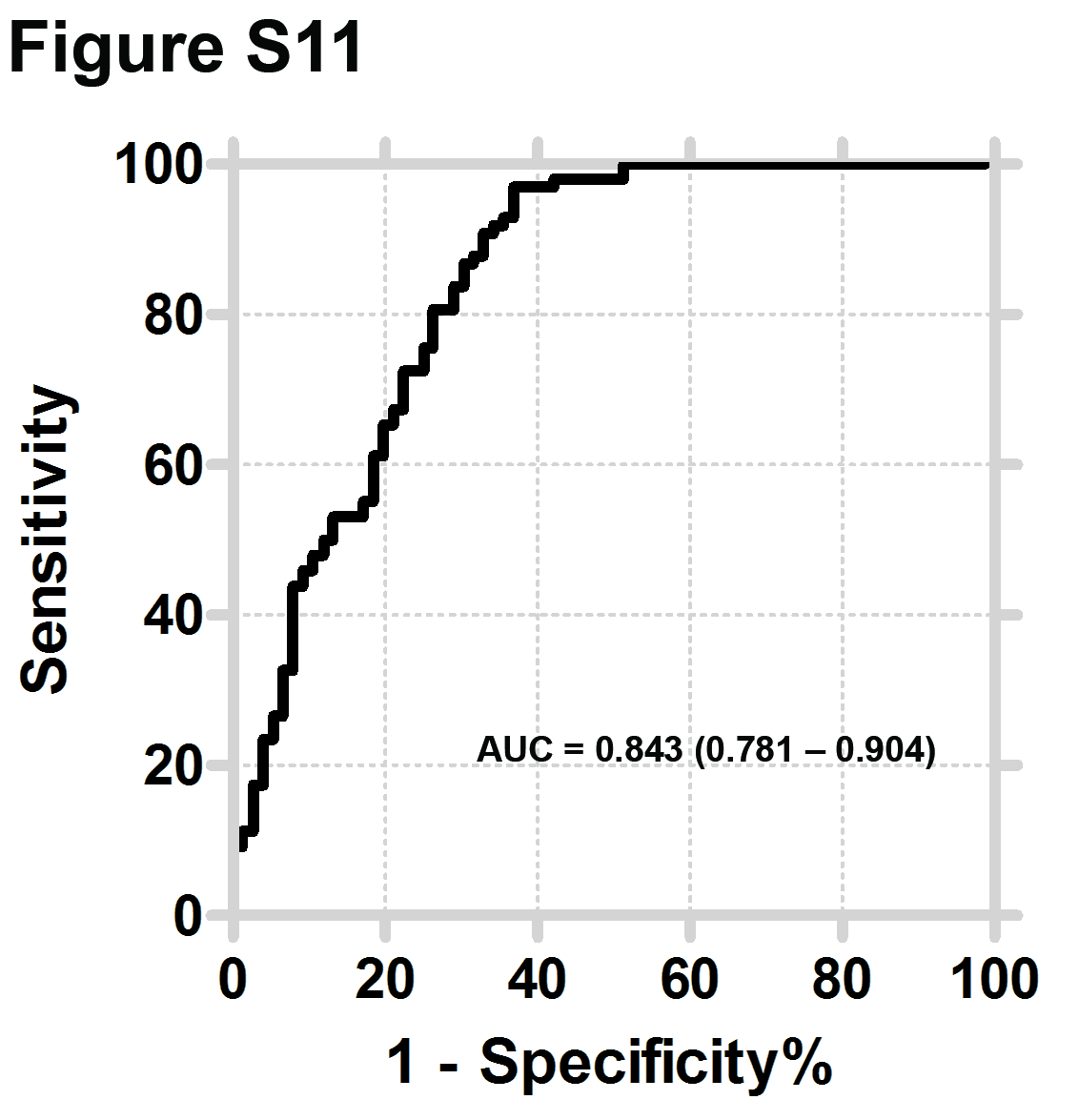

Supplement: Figure S11 — ROC analysis to determine the optimal percentage of methylated reference (PMR) for methylated NTRK3 that detects cancer specific levels of NTRK3 methylation. An ROC curve was constructed by plotting sensitivity vs. 1-specificity comparing adenocarcinomas (n = 76) to normal colon samples (n = 98). Area under the curve (AUC) for the sample set was 0.843 (95% CI0.781–0.904). (TIF) [file pgen.1003552.s011.tif]
