# Supplementary material for: NTRK3 Is a Potential Tumor Suppressor Gene Commonly Inactivated by Epigenetic Mechanisms in Colorectal Cancer
Source: PLoS Genet. 2013 Jul 11;9(7):e1003552. doi: 10.1371/journal.pgen.1003552 (PMC3708790; doi:10.1371/journal.pgen.1003552)
Supplement: Table S1 — Predicted effect of naturally occurring somatic mutations of NTRK3 in colorectal cancer. (DOCX) [file pgen.1003552.s012.docx]

**Table S1**. Predicted effect of naturally occurring somatic mutations of *NTRK3* in colorectal cancer

| **Mutation: AA change** | **BP change** | **PolyPhen** | **MutationTaster** | **Exome Variants** | **Conclusion** |
| --- | --- | --- | --- | --- | --- |
| G608S | G1822A | benign | disease causing | no | **uncertain** |
| I695V | A2083G | probably damaging | disease causing | no | **disease causing** |
| R731Q | G2192A | probably damaging | disease causing | no | **disease causing** |
| K732T | A2195C | probably damaging | disease causing | no | **disease causing** |
| L760I | C2278A | probably damaging | disease causing | no | **disease causing** |
| H599Y | C1795T | probably damaging | disease causing | no | **disease causing** |
| Polyphen: http://genetics.bwh.harvard.edu/pph2/ | | |  |  |  |
| Mutation Taster: http://www.mutationtaster.org/cgi-bin/MutationTaster/MutationTaster.cgi | | | | |  |
| Exome variants service: http://evs.gs.washington.edu/EVS/ | | |  |  |  |
| AA=amino acid; BP=basepair | | |  |  |  |
