# Supplementary material for: NTRK3 Is a Potential Tumor Suppressor Gene Commonly Inactivated by Epigenetic Mechanisms in Colorectal Cancer
Source: PLoS Genet. 2013 Jul 11;9(7):e1003552. doi: 10.1371/journal.pgen.1003552 (PMC3708790; doi:10.1371/journal.pgen.1003552)
Supplement: Table S2 — Clinical characteristics of the samples analyzed with the HumanMethylation450 array. (DOCX) [file pgen.1003552.s013.docx]

**Table S2**. Clinical characteristics of the samples analyzed with the HumanMethylation450 array

| **Sample ID** | **Histololgy** | **Age** | **Gender** | **Stage** |
| --- | --- | --- | --- | --- |
| 3325 | Normal colon | 63 | M |  |
| 3326 | Normal colon | 59 | M |  |
| 3327 | Normal colon | 50 | F |  |
| 3328 | Normal colon | 52 | M |  |
| 3329 | Normal colon | 59 | M |  |
| 3330 | Normal colon | 66 | M |  |
| CCR097 | Colon cancer | 23 | M | IV |
| CCR103 | Colon cancer | 68 | M | I |
| CCR109 | Colon cancer | 71 | F | IV |
| CCR115 | Colon cancer | 43 | F | III |
| CCR117 | Colon cancer | 79 | F | II |
| CCR143 | Colon cancer | 60 | M | III |
| CCR155 | Colon cancer | 58 | M | II |
| CCR159 | Colon cancer | 79 | F | IV |
