# Supplementary material for: NTRK3 Is a Potential Tumor Suppressor Gene Commonly Inactivated by Epigenetic Mechanisms in Colorectal Cancer
Source: PLoS Genet. 2013 Jul 11;9(7):e1003552. doi: 10.1371/journal.pgen.1003552 (PMC3708790; doi:10.1371/journal.pgen.1003552)
Supplement: Table S3 — Primer and Probe Sequences for NTRK3 reagents. (DOCX) [file pgen.1003552.s014.docx]

**Table S3**. Primer and Probe Sequences

| **NTRK3 MethyLight Primers and Probes** | |
| --- | --- |
| Forward | CGGCGTTCGCGATGGT |
| Reverse | ACCTTTAAAACGCCGAACGAT |
| Probe | TTAGACGTTGAAGGATTTTGTA |
| **NTRK3 Bisulfite Sequencing Primers** | |
| Forward | GATTTGGTGATTTTAGTATTATTTTT |
| Reverse | AAAAAAAACCTCTACCTTTAAAAC |
| **NTRK3 Plasmid Mutagenesis Primers** | |
| G608S |  |
| Forward | TCAAGTTCTATGGAGTGTGCAGCGATGGGGACCCCCTCATC |
| Reverse | GATGAGGGGGTCCCCATCGCTGCACACTCCATAGAACTTGA |
| I695V |  |
| Forward | GAGCGAATCTGCTAGTGAAGGTTGGGGACTTCGGCATGTCC |
| Reverse | GGACATGCCGAAGTCCCCAACCTTCACTAGCAGATTCGCTC |
| L760I |  |
| Forward | GAAAGCAGCCATGGTTCCAAATCTCAAACACGGAGGTCATT |
| Reverse | AATGACCTCCGTGTTTGAGATTTGGAACCATGGCTGCTTTC |
| **Mutant NTRK3 Plasmid Sequencing Primers** | |
| Primer 1 | ACTTCCGTCAGGGACACAAC |
| Primer 2 | CTCTCCCAAATGCTCCACAT |
| **NTRK3 shRNA Target Sequence** | |
| V3LHS_309053 | TGCAGCAAGACTGAGATCA |
| V2LMM_189598 | CGGTCCAAATTTGGAATGA |
| V2LMM_35392 | CGGCTGAATATTAAGGAGA |
| **shRNA Sequencing Primer** | |
| Primer 1 | GCATTAAAGCAGCGTATC |
