# Supplementary material for: NTRK3 Is a Potential Tumor Suppressor Gene Commonly Inactivated by Epigenetic Mechanisms in Colorectal Cancer
Source: PLoS Genet. 2013 Jul 11;9(7):e1003552. doi: 10.1371/journal.pgen.1003552 (PMC3708790; doi:10.1371/journal.pgen.1003552)
Supplement: Table S4 — Results of ROC analyses of the methylation levels in colon adenocarcinomas detected by MethyLight. (DOCX) [file pgen.1003552.s015.docx]

**Table S4**. Results of ROC analyses of the methylation levels in colon adenocarcinomas detected by MethyLight.

| **Tissue type** | **Cutoff**  **(PMR)** | **Sensitivity Estimate**  **(95% CI)** | **Specificity Estimate**  **(95% CI)** | **OR Estimate**  **(95% CI)** |
| --- | --- | --- | --- | --- |
| Adenocarcinoma (n=76)  Normal (n=98) |  |  |  |  |
|  | 4 | 0.934 (0.853 – 0.978) | 0.286 (0.199 – 0.386) | 5.7 (2.1 – 15.6) |
|  | 10 | 0.737 (0.623 – 0.831) | 0.786 (0.691 – 0.865) | 9.7 (4.8 – 19.4) |
|  | 13.7 | 0.671 (0.554 – 0.775) | 0.908 (0.833 – 0.957) | 19.0 (8.3 – 43.8) |
|  | 17.2^¶^ | 0.632 (0.513 – 0.739) | 0.939 (0.872 – 0.977) | 26.3 (10.2 – 67.9) |

^¶^ mean ± 2SD (standard deviation) based on the PMRs of the normal colon epithelium samples

AUC 0.843 (0.781 – 0.904)
