# Supplementary material for: NTRK3 Is a Potential Tumor Suppressor Gene Commonly Inactivated by Epigenetic Mechanisms in Colorectal Cancer
Source: PLoS Genet. 2013 Jul 11;9(7):e1003552. doi: 10.1371/journal.pgen.1003552 (PMC3708790; doi:10.1371/journal.pgen.1003552)
Supplement: Text S1 — Supplemental Methods. Description of methods used for studies whose data resulted in supplemental data and additional detailed description of methods used for studies in main text of manuscript. (DOCX) [file pgen.1003552.s016.docx]

**Supplemental Methods**

**Quantitative Methylation-Specific PCR (MethyLight)**

Quantitative methylation-specific PCR (qMSP; MethyLight) was performed using the ABI Prism 7700 detection system (Applied Biosystems). The primer/probe sequences for methylated *NTRK3* (NM_002530) were designed using ABI PrimerExpress software Version 5.0. The primer and probe sequences are in **Supplemental Data Table S3**. The reaction mix consisted of 0.45 µL primers (20 µM), 0.03 µL probe (100 µM), 1.5 M MgCl2, 200 pM dNTPs, HotStart Taq 1.5 U. The thermocycler conditions were as follows: 95 ^0^C for 10 minutes followed by 45 cycles of 30 seconds at 94 ^0^C, 30 seconds at 60 ^0^C; followed by 10 minutes at 72 ^0^C. AluC4 was used as a reference locus for normalization for input DNA following a previously described protocol ([30](#_ENREF_30)). SssI-treated DNA from the RKO CRC cell line was used as the methylated control reference sequence. All samples were run in triplicate in at least two independent reactions. The methylation status of the samples was determined using the PMR method described by Eads et al. ([31](#_ENREF_31)). Briefly, the percentage of fully methylated molecules at a specific locus was calculated by dividing the GENE:AluC4 ratio of a sample by the GENE: AluC4 ratio of SssI-treated RKO DNA and multiplying by 100. For the MethyLight results, samples with a C(t) value over 22 in the AluC4 assays were omitted in the final analyses based on recommended analysis methods (<http://www.nature.com/protocolexchange/protocols/46>). In order to determine the optimized cutoff of *NTRK3* PMR that discriminated the CRC from normal tissues, a receiver operator characteristic (ROC) curve was used. The optimal PMR value determined by the ROC analysis was used to determine whether the tissue samples were methylated or unmethylated.

**Determination of optimal *NTRK3* PMR**

We first generated an ROC curve and then used AUC analysis to determine the optimal percent of methylated reference (PMR) that discriminated cancer from normal colon (**Supplemental Data** **Figure S11**). The odds ratios (OR) for different PMR thresholds that detect cancer-specific methylated *NTRK3* in the colon cancers are shown in **Supplemental Data Table S4**.

**Predicting the effect of somatic mutations of *NTRK3* found in colorectal cancer**

Somatic mutations have been identified in human colorectal cancers in previous studies, and these mutations have been predicted to contribute to tumor formation ([15](#_ENREF_15), [16](#_ENREF_16)). We used publically available databases and software (PolyPhen and MutationTaster) to assess the potential effects of these mutations on the function of NTRK3. Using these programs we assessed the effects of the following somatic mutations of *NTRK3*: G608S (G1822A), I695V (A2083G), R731Q (G2192A), K732T (A2195C), L760I (C2278A) and H599Y (C1795T). If both databases predicted similar effects of the mutation, and there were no variants for the allele, we considered the prediction of a deleterious effect of the mutation on gene function highly reliable. (Table S1)
